# Cross-Infectivity of 11 Different Legume Species by 15 Native Rhizobia Isolated from African Soils

**DOI:** 10.3390/microorganisms13112463

**Published:** 2025-10-28

**Authors:** Lebogang J. Msiza, Titus Y. Ngmenzuma, Mustapha Mohammed, Sanjay K. Jaiswal, Felix D. Dakora

**Affiliations:** 1 Department of Crop Sciences, Tshwane University of Technology, Private Bag X680, Pretoria 0001, South Africa; lebogangjanemsiza@gmail.com (L.J.M.); ngmenzumatitus@gmail.com (T.Y.N.); 2 Department of Crop Science, University for Development Studies, Tamale P.O. Box TL 1882, Ghana; mmustapha@uds.edu.gh; 3 Department of Chemistry, Tshwane University of Technology, Private Bag X680, Pretoria 0001, South Africa; sanjaysiswa@gmail.com

**Keywords:** nodulation, shoot DM, N-fixed, percent relative symbiotic effectiveness, rhizobia

## Abstract

Selecting symbiotic rhizobia for use as inoculants in agriculture is a major challenge, though it is necessary for exploiting biological nitrogen fixation as an eco-friendly source of N in contrast to chemical N fertilizers which can pollute the environment. In addition to high symbiotic efficiency, bacterial strain ability to infect and effectively nodulate a wide range of host plants is also desired. Cross-infectivity studies are therefore important for identifying rhizobial strains that are highly effective with a broad host range. The legume/rhizobia symbiosis has the potential to contribute about 80% or more N to agricultural systems, thus providing a sustainable source of N in cropping systems. This study assessed the cross-nodulation, colony morphology, relative symbiotic effectiveness and N_2_ fixation of native rhizobial isolates from Africa that nodulate diverse legume species. The results showed that the rhizobial isolates differed significantly in symbiotic performance and relative symbiotic effectiveness. As a result, they differed markedly in nodulation and shoot DM induced in their host plants.

## 1. Introduction

Microbes, including bacteria, can affect the physical, chemical and biological properties of soil. A unique bacterial group called rhizobia can confer beneficial effects on legumes, including growth promotion by N_2_ fixation in root nodules [1,2]. These bacteria reduce atmospheric N_2_ to NH_3_ for their own use, though the surplus is released into host plant cells [3,4] where it is converted into organic N [5,6]. Of all the N_2_-fixing systems, the legume/rhizobia symbiosis is the most important for sustainable agriculture [7,8]. The formation of root nodules is host-specific, in that strains of rhizobia often nodulate only a limited range of leguminous plants [9,10,11]. Nodulation in legumes is mainly by bacteria belonging to the genera *Rhizobium*, *Bradyrhizobium*, *Azorhizobium*, *Sinorhizobium*, *Mesorhizobium*, etc. [2,12,13].

The growing interest in environmentally friendly, sustainable cropping systems has increased the use of rhizobial biofertilizers, which reduce the frequent use of chemical fertilizers in agriculture and their negative effect on the environment [14].

Most native soil rhizobia are either ineffective, partially effective or highly effective at fixing N_2_. It is therefore essential to identify, test and select highly effective local rhizobia before using them for inoculant production [15]. Screening for efficacy and competitiveness among indigenous N_2_-fixing isolates that are compatible with a broad host range should be a prerequisite for formulating rhizobial inoculants [16]. The aim of this study was to evaluate symbiotic effectiveness under glasshouse conditions.

## 2. Materials and Methods

### 2.1. Site Description

The experiments were conducted in a naturally lit glasshouse at the Tshwane University of Technology, Pretoria, South Africa, from October to December 2021 and January to April 2022.

### 2.2. Origin of the Legumes

Bambara groundnut (*Vigna subterranea*), common bean (*Phaseolus vulgaris*), jack bean (*Canavalia ensiformis*), winged bean (*Psophocarpus tetragonolobus*) and velvet bean (*Mucuna pruriens*) seeds were obtained from Etsala National Seed Co. Ltd., Malkerns, Swaziland; cowpea (*Vigna unguiculata*), soybean (*Glycine max*), mungbean (*Vigna radiata*), pigeonpea (*Cajanus caja*) and chickpea (*Cicer arietinum*) seeds from the International Institute of Tropical Agriculture, Mozambique (IITA-Mozambique, Maputo, Mozambique); Kersting’s bean seeds were obtained from the University for Development Studies (UDS) in Tamale, Ghana. The commercial inoculant strain used for cowpea, Bambara groundnut and Kersting’s bean were *Bradyrhizobium* strain CB756, common bean *Rhizobium leguminosarum* strain UD5 and soybean *Bradyrhizobium japonicum* strain WB74.

### 2.3. Authentication of Bacterial Isolates

Prior to planting, seeds of 11 test legume species (cowpea, Bambara groundnut, cowpea, Kersting’s groundnut, common bean, soybean, winged bean, velvet bean, pigeonpea, mungbean, jack bean and chickpea) were surface-sterilized by immersing them in 95% ethanol for 5 to 10 s, then in 3% sodium hypochlorite for 2–3 min, and rinsed six times with sterile distilled water [17]. The seeds were germinated in autoclaved sand contained in plastic pots (1200 cm^3^) covered with sterile non-absorbent cotton wool to avoid moisture loss and contamination. Three sterilized seeds were planted per pot and thinned out to one seedling per pot after germination. The legume seedlings were inoculated with 1 mL of broth culture (15 isolates) per isolate grown to exponential phase (10^6^–10^7^ cells/mL) using sterilized micro pipettes. The plants were irrigated with N-free nutrient solution [18], and when necessary, with sterile water. Uninoculated 5 mM KNO_3_-fed and commercial *Bradyrhizobium* inoculant were included as controls. Three replicate pots were used per isolate. The pots were arranged in a randomized block design. The plants were harvested at 60 days after planting for assessing root nodulation.

Each plant was separated into shoots and nodulated roots. The shoots were oven dried at 65 °C for 72 h weighed, ground into fine powder (0.50 mm sieve), and stored in plastic Ziploc bags prior to ^15^N isotopic analysis. The nodules were detached from the roots, washed to remove the sand, and stored on silica gel prior to bacterial isolation [19].

### 2.4. Bacteria Isolation from Root Nodule

The isolation of bacteria from root nodules of legume plants was performed as described by Somasegaran and Hoben [17]. Healthy nodules were selected and the bacteria isolated according to Ibny et al. [20]. The nodule suspensions were streaked onto yeast–mannitol agar (YMA) plates and incubated at 28 °C. The plates were monitored daily to record the time taken for colonies to appear. In instances where we did not obtain single colonies, re-streaking was performed to obtain single colonies.

### 2.5. Naming of the Isolates

The 15 rhizobial isolates used in this study were obtained from root nodules collected from Eswatini, Ghana and South Africa. Isolates were labeled based on the name of the institution where the study was carried out, the plant species from which the root nodule was sampled, the location where the nodule was collected, followed by a number for the isolate. For example, isolates from our lab at Tshwane University of Technology (TUT) sampled from cowpea plants *Vigna unguiculata* (Vu), and originating from South Africa (SA), would be TUTVuSA1.

### 2.6. Effectiveness of Rhizobial Isolates Under Glasshouse Conditions

The effectiveness of the rhizobial isolates was assessed sixty days after planting. The plants were separated into shoots, roots and nodules. The effectiveness of the rhizobial isolates was measured by nodule number, nodule dry weight and root and shoot dry weights. Nodules were counted, oven dried at 50 °C for 48 h and weighed. Plant shoots and roots were separately oven dried at 60 °C to a constant weight for 48 h and weighed. The percent relative effectiveness of the rhizobial isolates was calculated as follows [21]:

RSE = (Shoot dry matter inoculated plant/Shoot dry matter nitrate-fed plants) × 100

For each isolate < 35% RSE was considered ineffective; >35% RSE lowly effective; 50 to 80% RSE were moderately effective; and >80% RSE highly effective.

### 2.7. ^15^N/^14^N Isotopic Analysis

Analysis of shoot ^15^N/^14^N isotopic ratios was carried out at the Stable Isotope Laboratory, University of Cape Town, South Africa. Approximately 2.0 mg finely ground plant material was weighed into aluminum (Al) tin capsules to determine the %N and ^15^N values using a Carlo Erba NA1500 elemental analyzer (Fisons Instruments SpA, Strada, Rivoltana, Italy) coupled to a Finan MAT252 mass spectrometer (Finnigan, MAT CombH, Bremen, Germany) via a Conflo II Open-Split Device. The samples were run against an in-house reference material (Nasturtium) and the results normalized against and reported relative to atmospheric N as an international standard. The ^15^N natural abundance, expressed as δ (delta) notation, is the deviation of the ^15^N natural abundance of the sample from atmospheric N_2_ (0.36637 atom % ^15^N), and was calculated as follows [22,23]:

δN15=(N15/N14)sample−(N15/N14)atm(N15/N14)atm×1000

where ^15^N/^14^N_sample_ is the abundance ratio of ^15^N and ^14^N in the sample and ^15^N/^14^N_atm_ is the abundance ratio of ^15^N and ^14^N in the atmosphere.

### 2.8. Amount of N-Fixed in Shoots

The amount of N-fixed in shoots was calculated as follows:

N-fixed = shoot N content of inoculated plant − shoot N content of uninoculated control plants

### 2.9. Statistical Analysis

The data were subjected to an analysis of variance using the STATISTICA software, version 10.0 (StatSoft Inc., Tulsa, OK, USA). A one-way analysis of variance was performed to test significance, and the means separated using Duncan’s multiple range test at *p* ≤ 0.05. A correlation analysis was performed to determine any functional relationships between the measured parameters. Mean values (means ± se) with dissimilar letters in a column are significantly different at *p* < 0.05, ND = not determined.

## 3. Results

### 3.1. Plant Nodulation

Isolates TUTVuSA1, TUTVuSA2 and TUTVuSA3 were isolated from cowpea while TUTMgSA1, TUTMgSA2 and TUTMgSA3 were isolated from Kersting’s groundnut. TUTVsES1, TUTVsES2 and TUTVsES3 were isolated from Bambara groundnut, TUTPvES1, TUTPvES2 and TUTPvES3 from common bean and TUTGmGH1, TUTGmGH2 and TUTGmGH3 from soybean. The cross-inoculation experiment involved two genotypes of each of the test legumes (except for winged bean). The experiment was carried out using one genotype of each legume in 2021 and repeated using a different genotype for each legume in 2022 (Tables S1 and S2).

**Figure 1:**
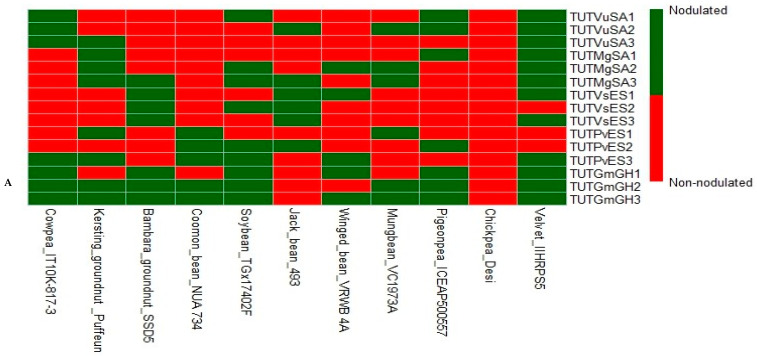

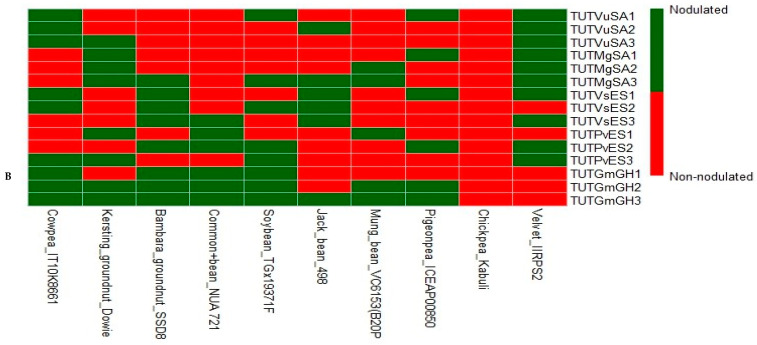
Heatmap showing nodulation of diverse legume species by native rhizobial isolates planted under glasshouse conditions in 2021 (**A**) and 2022 (**B**).

In 2021, cowpea cv. IT10K-817-3 was effectively nodulated by its own compatible rhizobia isolates TUTVuSA1, TUTVuSA2 and TUTVuSA3 together with strains TUTGmGH1, TUTGmGH2 and TUTGmGH3 from soybean (Figure 1A). Bambara groundnut landrace SSD5 was nodulated by its own isolates TUTVsES1, TUTVsES2 and TUTVsES3, as well as by isolates TUTGmGH1, TUTGmGH2 and TUTGmGH3 from soybean. Kersting’s groundnut landrace Puffeun was also nodulated by its own rhizobia TUTMgSA1, TUTMgSA2 and TUTMgSA3, as well as by isolate TUTVuSA3 from cowpea, TUTPvES1 and TUTPvES3 from common bean and TUTGmGH2 and TUTGmGH3 from soybean. Common bean cv. NUA 734 was effectively nodulated by bean isolates TUTPvES1, TUTPvES2 and TUTPvES3 and soybean isolates TUTGmGH2 and TUTGmGH3, though they were ineffective. Soybean genotype TGX1740-2F was effectively nodulated soybean isolates TUTGmGH1, TUTGmGH2 and TUTGmGH3, as well as by isolate TUTMgSA3 from Kersting’s groundnut and TUTVsES2 from Bambara groundnut, TUTPvES2 and TUTPvES3 from common bean and TUTVuSA1 from cowpea. Winged bean was nodulated by isolates TUTMgSA2 from Kerting’s bean, TUTVsES1 from Bambara groundnut, TUTPvES3 from common bean and TUTGmGH1, TUTGmGH2 and TUTGmGH3 from soybean; however, isolate TUTPvES3 was ineffective. Although isolates TUTVuSA1, TUTVuSA2 and TUTVuSA3 from cowpea, TUTMgSA1, TUTMgSA2 and TUTMgSA3 from Kersting’s groundnut, TUTVsES1 and TUTVsES3 from Bambara groundnut, TUTPvES2 and TUTPvES3 from common bean and TUTGmGH1, TUTGmGH2 and TUTGmGH3 from soybean nodulated velvet bean genotype IIHR PS 1, only five isolates were effective, which included TUTVuSA1, TUTPvES2, TUTPvES3, TUTVsES1 and TUTMgSA3. Jack bean genotype Accession 493 was effectively nodulated by TUTVuSA2, TUTMgSA3, TUTVsES1, TUTVsES2, TUTVsES3 and TUTPvES2. Pigeonpea (cv. ICEAP500557) was effectively nodulated by TUTVuSA1, TUTMgSA1, TUTPvES2, TUTGmGH1, TUTGmGH2 and TUTGmGH3, and ineffectively by isolate TUTVuSA2. Mungbean (cv.VC1973A) was also effectively nodulated by TUTMgSA2, TUTMgSA3, TUTGmGH2, TUTGmGH3 and TUTPvES1. In contrast, chickpea genotype Desi failed to form root nodules with all test rhizobial isolates (Figure 1A).

In 2022, cowpea (cv. IT10K-866-1) was effectively nodulated by isolates TUTGmGH1, TUTGmGH2, TUTGmGH3 and TUTPvES3 from common bean and TUTVsES1 and TUTVsES2 from Bambara groundnut, as well as by its own symbionts TUTVuSA1, TUTVuSA2 and TUTVuSA3 (Figure 1B). Bambara groundnut was also effectively nodulated by its own isolates TUTVsES1, TUTVsES2 and TUTVsES3, as well as by the three soybean isolates (TUTGmGH1, TUTGmGH2 and TUTGmGH3), TUTMgSA3 from Kersting’s groundnut and TUTPvES2 from common bean and the commercial inoculant strain. Isolates TUTVuSA3 (cowpea), TUTPvES1 and TUTPvES3 (common bean), as well as TUTGmGH2 and TUTGmGH3 (soybean), effectively induced nodulation in Kersting’s groundnut (landrace Dowie) in addition to its own symbionts (TUTMgSA1, TUTMgSA2 and TUTMgSA3) and the inoculant strain. Common bean (cv. NUA 721) was effectively nodulated by isolates TUTGmGH1, TUTGmGH2 and TUTGmGH3 from soybean as well as by its own isolates TUTPvES1, TUTPvES2 and TUTPvES3 and the commercial strain. Soybean (cv. TGX1937-1F) was effectively nodulated by its own isolates (TUTGmGH1, TUTGmGH2 and TUTGmGH3), as well as by isolates TUTVuSA1, TUTMgSA3, TUTVsES2 and TUTPvES3 from different legumes and the inoculant strain. Although velvet bean (cv.IIHR PS 2) was nodulated by isolates TUTVuSA1, TUTVuSA2, TUTVuSA3, TUTMgSA1, TUTMgSA2, TUTMgSA3, TUTVsES3, TUTPvES2, TUTPvES3, TUTGmGH1, TUTGmGH2 and TUTGmGH3, only six microsymbionts were effective, which included TUTVuSA1, TUTVuSA2, TUTPvES2, TUTPvES3, TUTVsES3 and TUTGmGH3. Although jack bean (Accession 498) formed root nodules with isolates TUTVuSA2, TUTMgSA3, TUTVsES1, TUTVsES2, TUTVsES3, TUTGmGH3 and TUTPvES2, only two strains (TUTVuSA2 and TUTVsES2) were effective. Pigeonpea (cv. ICEAP00850) was effectively nodulated by isolates TUTVuSA1, TUTMgSA1, TUTVsES1, TUTPvES2, TUTGmGH2 and TUTGmGH3. Similarly, mungbean (cv. VC6153 (B-20P) was effectively nodulated by isolates TUTMgSA2, TUTMgSA3, TUTPvES1, TUTGmGH2 and TUTGmGH3 while chickpea (cv. Kabuli) failed to form root nodules with all test isolates (Figure 1B).

### 3.2. Nodule Number and Nodule DM of Test Legumes

#### 3.2.1. Cowpea (*Vigna unguiculata*)

**Figure 2:**
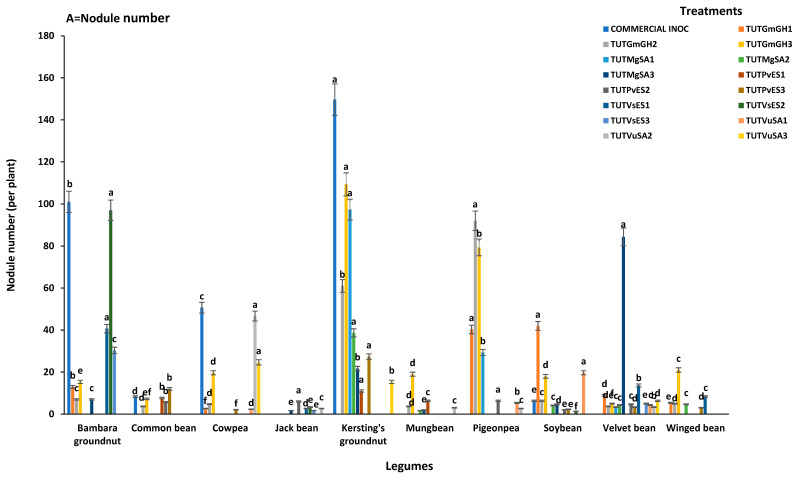

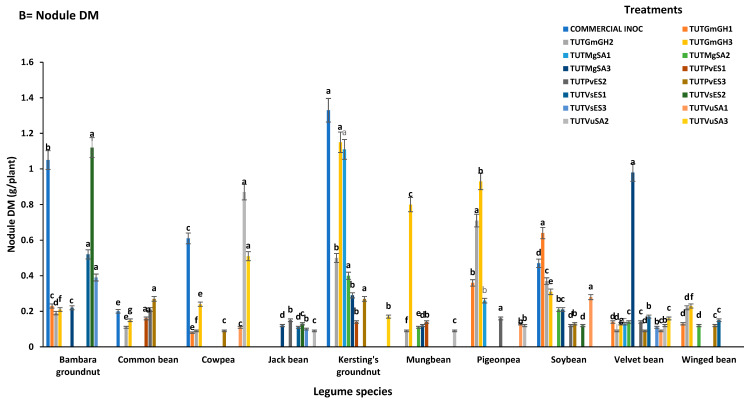
Nodule number and nodule dry matter of legume species used in cross-infectivity test in the glasshouse in 2021: (**A**) = nodule number and (**B**) = nodule DM. Bars with dissimilar letters are significantly different at *p* < 0.05. Missing bars are denoted as zero nodulation.

**Figure 3:**
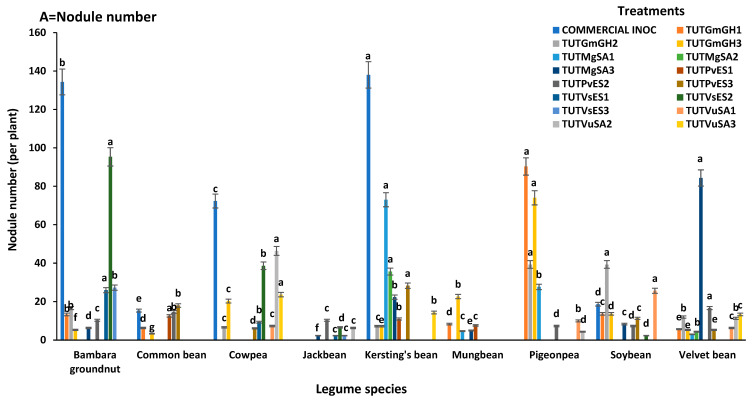

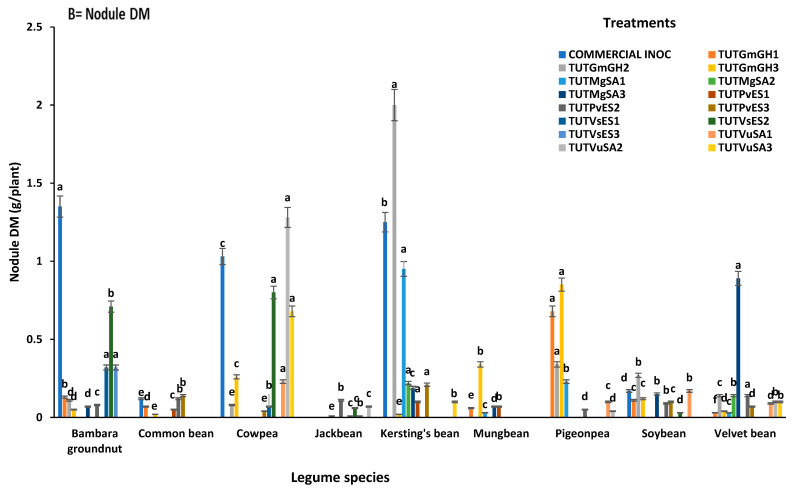
Nodule number and nodule dry matter of legume species used in cross-infectivity test in the glasshouse in 2022: (**A**) = nodule number and (**B**) = nodule DM. Bars with dissimilar letters are significantly different at *p* < 0.05. Missing bars are denoted as zero nodulation.

With cowpea (cv.IT10K-817-3) as a homologous host, the bacterial isolates induced different levels of nodulation. Nodule number per plant varied significantly and ranged from 2 to 50 (Table S3). Nodule DM was highest in the plants inoculated with isolate TUTVuSA2 (0.87 g.plant^−1^), followed by those inoculated with the commercial inoculant strain (0.61 g.plant^−1^) and TUTVuSA3 (0.51 g.plant^−1^), while the plants inoculated with isolate TUTGmGH1 (0.08 g.plant^−1^) produced the lowest nodule DM (Figure 2A,B). With cowpea (cv. IT10K-866-1) as the homologous host, nodule number and nodule DM per plant also differed among the plants inoculated with the various rhizobial isolates (Table S4). Nodule number ranged from 6 to 72 per plant, with the commercial strain eliciting the highest nodule number (72 per plant). Cowpea plants inoculated with the commercial inoculant strain recorded the highest nodule number per plant, while those inoculated with isolate TUTVuSA2 produced the highest nodule DM (Figure 3A,B).

#### 3.2.2. Bambara Groundnut (*Vigna subterranea*)

Bambara groundnut nodulation by the bacterial isolates with landrace SSD5 as a homologous host (Table S3). The highest nodule number (101 per plant) was recorded by the plants inoculated with the commercial inoculant, followed by those inoculated isolate TUTVsES2 (97 per plant), while the plants inoculated with isolate TUTGmGH1 recorded the lowest nodule number (13 per plant). However, in 2021, nodule DM ranged from 0.19 to 1.12 g.plant^−1^, with isolates TUTVsES2 and TUTVsES1 showing the highest nodule mass, and TUTGmGH2 the lowest (Figure 2A,B). With Bambara groundnut landrace SSD8 as a homologous host, nodule numbers varied significantly and ranged from 6 to 134 nodules per plant. The commercial inoculant elicited the highest nodule number per plant and isolate TUTMgSA3 the lowest. As a result, plants inoculated with the commercial inoculant produced the highest nodule DM, while those inoculated with isolate TUTMgSA3 produced the lowest (Figure 3A,B).

#### 3.2.3. Kersting’s Groundnut (*Macrotyloma geocarpum*)

The test rhizobial isolates induced different levels of nodulation in Kersting’s groundnut using two homologous hosts (Tables S3 and S4). With landrace Puffeun, the highest nodule number was elicited by the commercial inoculant (149 per plant), TUTGmGH3 (109 per plant) and TUTMgSA1 (97 per plant), while the lowest was by isolate TUTPvES1 (11 per plant). With landrace Puffeun as the host plant, nodule DM ranged from 0.14 to 1.33 g.plant^−1^, with isolate TUTGmGH3 producing the highest and TUTPvES1 the lowest (Figure 2A,B). With landrace Dowie as the homologous host, nodule number and nodule DM also varied significantly (Table S4), with the commercial inoculant eliciting the highest nodule number (138 per plant) and isolate TUTVuSA3 the lowest (14 per plant). The nodule DM also ranged from 1.25 g.plant^−1^ by the commercial inoculant to 0.10 g.plant^−1^ for isolates TUTPvES1 and TUTVuSA3 (Figure 3A,B).

#### 3.2.4. Common Bean (*Phaseolus vulgaris*)

There were no significant differences in the nodule number of common bean using cv. NUA 734 as a homologous host; here nodule numbers ranged from 3 to 8 per plant (Table S3). However, with cv. NUA 721 as the host plant, nodule number and nodule DM also differed among the test isolates (Table S4). Nodule number ranged from 3 to 18 per plant, while nodule DM ranged from 0.02 g.plant^−1^ for plants isolated with isolate TUTGmGH3 to 0.14 g.plant^−1^ for those inoculated with isolate TUTPvES3 (Figure 3A,B).

#### 3.2.5. Soybean (*Glycine max*)

Nodule number and nodule DM induced by the isolates differed when soybean cv. TGX1740-2F was used as the host plant (Table S3). Isolate TUTGmGH1 elicited much higher nodule numbers per plant (42) compared to the other isolates and commercial inoculant. As a result, isolate TUTGmGH1 produced much higher nodule DM compared to the other isolates (Figure 2A,B). With cv. TGX1937-1F as a homologous host, isolate TUTGmGH2 (39 per plant), TUTVuSA1 (25 per plant) and the commercial inoculant (18 per plant) produced higher nodule numbers. Nodule DM induced by the isolates ranged from 0.03 to 0.27 g.plant^−1^ (Figure 3A,B).

#### 3.2.6. Winged Bean (*Psophocarpus tetragonolobus*)

With winged bean as the host plant, nodule number per plant varied significantly, with isolate TUTGmGH3 eliciting the highest nodule number (21.0) and isolate TUTPvES3 the lowest of (3.0 per plant) (Table S3). There was no significant difference in nodule DM induced by the test isolates, values ranging from 0.12 g.plant^−1^ to 0.23 g.plant^−1^ (Figure 2A,B).

#### 3.2.7. Velvet Bean (*Mucuna pruriens*)

Using velvet bean (cv. IIHR PS 1) in 2021 as the host, nodule number and nodule DM varied markedly among the isolates, with TUTMgSA3 producing the highest nodule number (84) and TUTPvES3 the lowest (3 per plant). Nodule DM ranged from 0.09 to 0.98 g.plant^−1^, with isolate TUTMgSA3 recording the highest nodule DM (Figure 2A,B). With velvet cv. IIHR PS 2, nodule number differed significantly and ranged from 3 to 84 per plant (Table S4). Isolate TUTMgSA1 produced the lowest nodule number (3 per plant) and TUTMgSA3 the highest (84 per plant). Isolate TUTMgSA3 recorded the highest nodule DM (0.89 g.plant^−1^) and those inoculated with isolate TUTMgSA1 and TUTGmGH1 recorded as the lowest value (0.03) (Figure 3A,B).

#### 3.2.8. Jack Bean (*Canavalia ensiformis*)

With Jack bean cv.Accession 493 in 2021, the test rhizobial isolates induced different levels of nodulation on jack bean genotype. Nodule number ranged from 1 to 6 per plant, with isolate TUTPvES2 producing the highest nodule number per plant (6 per plant), and TUTMgSA3 and TUTVsES3 the lowest (1 per plant). However, isolate TUTPvES2 recorded the highest nodule (0.15 g.plant^−1^) and TUTVsES3 recorded the lowest (0.09 g.plant^−1^) (Figure 2A,B). With cv.Accession 498 in 2022, jack bean nodule number and nodule DM similarly varied among the isolates (Table S4). Nodule number per plant ranged from 2.33 to 10.33, with isolate TUTPvES2 eliciting the highest nodule number, and isolates TUTMgSA3, TUTVsES2 and TUTVsES3 the lowest. However, TUTGmGH3 produced greater nodule DM compared to the other isolates, with the lowest nodule DM by TUTGmGH1 (Figure 3A,B).

#### 3.2.9. Pigeonpea (*Cajanus cajan*)

For the pigeonpea genotype ICEAP500557 in 2021, root nodulation differed among the bacterial isolates; the highest nodule number per plant was recorded by isolate TUTGmGH2 (92 per plant) followed by TUTGmGH3 (79 per plant), and the lowest by isolate TUTVuSA2 (2 per plant) (Figure 2A,B). However, with the pigeonpea genotype ICEAP00850 in 2022, nodule number varied significantly and ranged from 4 to 90 per plant. Isolate TUTGmGH3 produced the highest nodule DM, followed by TUTGmGH1 with TUTVuSA2 the lowest (Figure 3A,B).

#### 3.2.10. Mungbean (*Vigna radiata*)

Using mungbean genotype (cv.VC1973A) in 2021, nodule number and nodule DM differed significantly among the isolates (Table S3). For example, isolate TUTGmGH3 recorded the highest nodule number per plant (19) compared to other isolates, with TUTMgSA2 recording the lowest (1 per plant). While nodule DM was lower with isolate TUTGmGH2 (0.09 g.plant^−1^), TUTGmGH3 produced the highest (0.80 g.plant^−1^) compared to the other isolates (Figure 2A,B). However, with genotype VC6153 (B-20P) in 2022, nodule number and nodule DM varied significantly (Table S4). Nodule number ranged from 4 to 22 per plant and nodule DM from 0.03 to 0.34 mg.plant^−1^ (Figure 3A,B).

### 3.3. Shoot DM of Test Legumes Species Inoculated with Different Rhizobial Isolates

#### 3.3.1. Cowpea (*Vigna unguiculata*)

With cowpea, shoot DM showed significant differences among the test isolates (Table 1 and Table 2). Using genotype IT10K-817-3 as the homologous host in 2021, the nitrate-fed plants and the commercial strain produced the highest shoot DM (3.83 g.plant^−1^ and 3.73 g.plant^−1^ respectively), followed by isolates TUTVuSA1 (3.4 g.plant^−1^), TUTVuSA2 (3.5 g.plant^−1^) and TUTVuSA3 (2.57 g.plant^−1^), which were originally obtained from cowpea. Isolate TUTGmGH2 produced the lowest shoot biomass (0.43 g.plant^−1^). However, with cowpea genotype IT10K-866-1 as the homologous host in 2022, isolate TUTVuSA1 produced the most shoot DM, followed by isolate TUTVuSA2 and TUTVuSA3, with TUTGmGH3 producing the lowest (Table 2).

#### 3.3.2. Bambara Groundnut (*Vigna subterranea*)

With Bambara groundnut landrace SSD5 as the homologous host in 2021, shoot DM differed significantly among plants inoculated with the test isolates (Table 1). Nitrate-fed plants and the commercial strain, as well as isolates TUTVsES1, TUTVsES2 and TUTVsES3 obtained originally from Bambara groundnut, induced greater shoot DM accumulation (Table 1). Using landrace SSD8 as the host plant, plant inoculated with isolate TUTVsES2 accumulated greater shoot DM (3.83 g.plant^−1^), followed by the commercial inoculant strain (3.87 g.plant^−1^), and isolates TUTVsES1 (3.83 g.plant^−1^), the nitrate-fed plants (3.03 g.plant^−1^) and TUTVsES3 (2.50 g.plant^−1^). Isolate TUTPvES2 produced the lowest shoot DM (0.73 g.plant^−1^) (Table 2).

#### 3.3.3. Kersting’s Groundnut (*Macrotyloma geocarpum*)

Shoot biomass revealed highly significant differences among the test isolates when Kersting’s groundnut landrace Puffeun was used as the homologous host in the cross-nodulation assay. Shoot biomass ranged from 0.87 to 2.20 g.plant^−1^ in 2021 (Table 1). With landrace Dowie as homologous host, shoot biomass ranged from 0.97 to 3.20 g.plant^−1^ in 2022 (Table 2).

#### 3.3.4. Common Bean (*Phaseolus vulgaris*)

With common bean genotypes NUA 734 as host plants, isolates TUTVsES1, TUTPvES1, TUTPvES2 and TUTPvES3, as well as the commercial strain, showed greater shoot DM than the other isolates (Table 1), However, with cv. NUA 721 as the host plant, isolate TUTPvES2 and TUTPvES1 produced the next highest shoot DM (3.03 g.plant^−1^) after the commercial strain (3.30 g.plant^−1^). Isolate TUTGmGH3 showed the lowest shoot DM (2.10 g.plant^−1^) (Table 2).

#### 3.3.5. Soybean (*Glycine max*)

With soybean genotype TGX1740-2F as the homologous host, isolates TUTGmGH1, TUTGmGH2 and TUTGmGH3 as well as the commercial strain accumulated greater shoot DM than the other test isolates (Table 1). With cv. TGX1937-1F as the homologous host, shoot DM ranged from 0.93 to 2.80 g.plant^−1^, with the three strains again inducing the highest shoot DM (Table 2).

#### 3.3.6. Winged Bean (*Psophocarpus tetragonolobus*)

Winged bean showed highly significant differences in shoot biomass accumulation when nodulated by the test isolates (Table 1). Isolate TUTVsES3 originally obtained from the root nodule of Bambara groundnut in Eswatini induced the highest shoot DM (3.10 g.plant^−1^) in winged bean (Table 1).

#### 3.3.7. Velvet Bean (*Mucuna pruriens*)

With velvet bean cv. IIHR PS 1 in 2021 as the host, isolate TUTVsES2 from Bambara groundnut accumulated the highest shoot biomass compared to other isolates. TUTPvES2 stimulated the least biomass production. Using velvet bean cv. IIHR PS 2 in 2022 revealed marked differences in shoot DM among the test isolates. Here, TUTVuSA3 accumulated the highest shoot DM (4.00 g.plant^−1^), followed by TUTVuSA1 (3.77 g.plant^−1^), nitrate-feeding (3.63 g.plant^−1^) and TUTGmGH1 (3.60 g.plant^−1^) (Table 2).

#### 3.3.8. Jack Bean (*Canavalia ensiformis*)

When the jack bean Accession 493 was used as the host plant in 2021, isolate TUTMgSA2 elicited the highest shoot DM (Table 1). The lowest shoot DM was recorded in the plants inoculated with isolate TUTVsES2 (Table 1). Using Accession 498 as the host plant in 2022, the shoot DM ranged from 3.37 g.plant^−1^ in the plants inoculated with isolate TUTVuSA2 to 4.87 g.plant^−1^ in the nitrate-fed plants (Table 2).

#### 3.3.9. Pigeonpea (*Cajanus cajan*)

With cv. ICEAP500557 in 2021 as the host, the shoot DM differed significantly among the test isolates, with TUTVsES1 and TUTPvES2 inducing a greater increase in the shoot DM of pigeonpea (Table 1). Shoot DM ranged from 0.63 to 1.93 g.plant^−1^ and whole-plant DM from 1.13 to 3.20 g.plant^−1^ (Table 2).

#### 3.3.10. Mungbean (*Vigna radiata*)

Using mungbean cv.VC1973A in 2021, shoot DM differed among the isolates, with TUTPvES3 inducing much greater shoot DM. With mungbean cv. VC6153 (B-20P), the shoot DM also differed significantly among isolates, with shoot biomass ranging from 0.43 to 1.33 g.plant^−1^ (Table 2).

### 3.4. Shoot δ^15^N, %N and N-Fixed of Diverse Legume Species Inoculated with Native Rhizobial Isolates

#### 3.4.1. Cowpea (*Vigna unguiculata*)

With cowpea cv. IT10K-817-3 as a host plant in 2021, isolate TUTGmGH2 (4.96‰), followed by TUTVsES1 (3.72‰), TUTVuSA1 (2.58‰) and TUTVsES2 (2.03‰) recorded much greater shoot δ^15^N values compared to the other isolates. In contrast, isolate TUTVuSA3 recorded the lowest shoot δ^15^N. The isolates with high δ^15^N values revealed low shoot N concentrations and vice versa. Isolate TUTVuSA3, followed by the commercial strain and TUTVuSA2 showed the highest amount of N-fixed, and isolate TUTVsES1 the lowest (Table 1). With cowpea cv. IT10K-866-1 as the host plant, isolate TUTVuSA3 (4.96‰) and TUTVuSA2 (3.63‰) recorded the highest δ^15^N values and therefore showed the lowest N concentration. The commercial inoculant strain exhibited the lowest δ^15^N value and hence the highest N concentration. The N-fixed values ranged from 8.86 mg.plant^−1^ for TUTGmGH3 to 90.43 mg.plant^−1^ for the commercial inoculant (Table 2).

#### 3.4.2. Bambara Groundnut (*Vigna subterranea*)

With Bambara groundnut landrace SSD5, shoot δ^15^N values ranged from 0.23‰ to 1.51‰ (Table 1). Isolates TUTGmGH2 and TUTGmGH3 recorded the lowest %N and TUTVsES1 and TUTMgSA3 recorded the highest after the commercial inoculant (3.15%) and nitrate-fed plants (2.99%). Isolate TUTVsES1 (126.89 mg.plant^−1^) and the commercial inoculant (107.51 mg.plant^−1^) produced a much higher amount of N-fixed compared to the other isolates (Table 1). With landrace SSD8 as the host plant in 2022, there was a marked variation in δ^15^N, with values ranging from 0.20 for TUTGmGH1 to 0.69‰ for TUTVsES3. Isolates TUTVsES3 (2.95%) and TUTVsES1 (2.26%) showed the highest N concentration, and isolate TUTPvES2 (1.29%) the lowest. The amount of N-fixed was highest for isolate TUTVsES1 (73.25 mg.plant^−1^), followed by TUTVsES2 (71.75 mg.plant^−1^), TUTVsES3 (69.71 mg.plant^−1^) and the commercial strain (63.78 mg.plant^−1^). N-fixed was, however, lowest for isolate TUTPvES2 (Table 2).

#### 3.4.3. Kersting’s Groundnut (*Macrotyloma geocarpum*)

With Kersting’s groundnut landrace Puffeun as the host plant, shoot δ^15^N differed significantly among the nodulating isolates, with values ranging from 0.21‰ for plants inoculated with isolate TUTMgSA3 to 1.26‰ for TUTGmGH1. Shoot % N ranged from 0.98% for TUTGmGH3 to 2.94% for TUTMgSA3. The amount of N-fixed was highest for the plants that were treated with the inoculant strain (97.62 mg.plant^−1^), followed by TUTMgSA3 and TUTMgSA1, and lowest for TUTGmGH1 (Table 1). 

With Kersting’s groundnut landrace Dowie as the host plant, shoot δ^15^N again differed among the isolates used to inoculate Kersting’s groundnut. Isolate TUTGmGH2 (2.46‰) showed the highest δ^15^N and TUTGmGH3 the lowest. As a result, plants with the low δ15N values recorded high N concentration, while the high δ^15^N reflected low N concentration. Fixed-N was highest for TUTMgSA3 (54.04 mg.plant^−1^), followed by TUTMgSA1 (48.09 mg.plant^−1^) and TUTMgSA2 (31.13 mg.plant^−1^) (Table 2).

#### 3.4.4. Common Bean (*Phaseolus vulgaris*)

With common bean cv. NUA 734 as the host plant, shoot δ^15^N ranged from 2.30‰ to 4.08‰ for the plants inoculated with isolate TUTPvES3 and inoculant strain, respectively. Isolate TUTPvES3 (3.01%) and the commercial strain (2.93%) recorded the highest N concentration. The inoculant strain (171.87 mg.plant^−1^), TUTPvES3 (90.21 mg.plant^−1^) and TUTPvES2 (65.77 mg.plant^−1^) produced a markedly greater amount of N-fixed than the other isolates (Table 1). With common bean cv. NUA 721 as a host plant, δ^15^N values were lowest for isolate TUTPvES2, followed by TUTPvES3. Shoot N concentration was highest for TUTPvES3, followed by the commercial strain (Table 2). The amount of N-fixed was highest for the inoculant strain, followed by TUTPvES1, and lowest for TUTGmGH2.

#### 3.4.5. Soybean (*Glycine max*)

With soybean cv. TGX1740-2F as the host plant, isolates TUTMgSA3 and TUTPvES3 elicited the highest shoot δ^15^N, and TUTPvES2 the lowest. Isolate TUTGmGH1 showed the highest shoot %N (3.61%) followed by TUTVuSA1 (3.17%), with the lowest values found in the nitrate-fed plants. Fixed-N was highest with isolates TUTGmGH2 and TUTGmGH3, and lowest with isolate TUTPvES3 (Table 1). With soybean cv. TGX1937-1F, isolate TUTMgSA3 (3.79‰), followed by TUTVsES2 (2.05‰) and TUTVuSA1 (1.43‰) revealed the highest shoot δ^15^N, with TUTPvES3 as the lowest. The shoot N concentration was highest for TUTGmGH1, followed by TUTVuSA1 and TUTPvES2. The amount of N-fixed was highest with isolate TUTGmGH1, followed by TUTGnGH2, and TUTVuSA1, and lowest with TUTMgSA3 and TUTVsES2 (Table 2).

#### 3.4.6. Winged Bean (*Psophocarpus tetragonolobus*)

With winged bean in 2021, shoot δ^15^N was lowest in the plants inoculated with isolate TUTGmGH2 and higher in the plants inoculated with the other isolates (Table 1). As a result, the plants inoculated with isolate TUTGmGH2 recorded the highest %N while the plants inoculated with isolate TUTMgSA2 had the lowest %N. N-fixed was highest in the plants inoculated with isolate TUTGmGH1, followed by the plants inoculated with isolate TUTGmGH2; here, the lowest N-fixed was recorded in the plants inoculated with isolate TUTMgSA2 (Table 1).

#### 3.4.7. Velvet Bean (*Mucuna pruriens*)

With velvet bean cv. IIHR PS 1 as a host, the shoot δ^15^N values ranged from 0.07‰ to 1.06‰, though there were no significant differences among the isolates. Isolates TUTMgSA2 and TUTVsES1 (1.38%) induced the higher N concentration than the others. Isolate TUTVuSA3 (50.12%) recorded the highest amount of N-fixed and TUTPvES2 (22.58%) the lowest (Table 1). With cv. IIHR PS 2, isolates TUTPvES2 (0.90‰), TUTVuSA3 (0.83‰), TUTMgSA3 (0.70‰) and TUTVsES2(0.69‰) showed the highest shoot δ^15^N. Shoot N concentrations were similar for all isolates. However, the amount of N-fixed ranged from 17.50 mg.plant^−1^ to 55.84 mg.plant^−1^ (Table 2).

#### 3.4.8. Jack Bean (*Canavalia ensiformis*)

With jack bean cv. Accession 493, isolate TUTVsES1 (3.38‰) followed by TUTPvES2 (3.32‰) and TUTMgSA3 (3.07‰) elicited the highest shoot δ^15^N values. However, N concentration was similar for all inoculated plants. N-fixed was highest for TUTVuSA2, TUTVsES2 and TUTVsES3 and lowest for TUTPvES2 (Table 1). With Accession 498 as host, shoot δ^15^N varied significantly among the isolates, with values ranging from 0.32‰ to 3.85‰. Isolate TUTGmGH3 (3.43%) recorded the highest %N and TUTVuSA1 (1.23%) the lowest. Isolate TUTGmGH3 recorded the highest amount of N-fixed (Table 2).

#### 3.4.9. Pigeonpea (*Cajanus cajan*)

With pigeonpea cv. ICEAP500557 as the host plant, shoot δ^15^N was greater with isolate TUTMgSA1 and lowest with TUTPvES2. Isolate TUTGmGH3 (2.47%) followed by TUTGmGH2 (1.53%) and TUTMgSA1 (1.32%) showed much greater shoot N concentration. Isolate TUTGmGH2 (15.99 mg.plant^−1^) recorded the highest amount of N-fixed and TUTVuSA1 (8.82 mg.plant^−1^) the lowest (Table 1). With pigeonpea cv. ICEAP00850, shoot δ^15^N, values ranged from 1.06‰ for TUTPvES2 to 3.64‰ for isolate TUTMgSA1. Isolate TUTGmGH3 (2.34%) recorded the highest N level and nitrate-fed plants the lowest. Isolate TUTGmGH3 (22.04 mg.plant^−1^) also recorded the highest amount of N-fixed (Table 2).

#### 3.4.10. Mungbean (*Vigna radiata*)

With mungbean cv. VC1973A, isolate TUTGmGH1 (4.16‰) recorded the highest shoot δ^15^N and TUTGmGH3 the lowest. The shoot N% ranged from 1.26 for TUTMgSA3 to 3.36 for TUTGmGH1. Isolate TUTGmGH3 recorded the highest amount of N-fixed compared to the other isolates (Table 1). With cv. VC6153 (B-20P), isolate TUTMgSA3 (4.40‰), followed by TUTMgSA2 (2.78‰), and TUTPvES1 (2.75‰) recorded higher shoot δ^15^N than the other isolates. Isolate TUTGmGH3 exhibited the lowest δ^15^N and hence the highest %N, as well as the amount of N-fixed (Table 2).

### 3.5. Relative Symbiotic Effectiveness (RSE) of Isolates

#### 3.5.1. Cowpea (*Vigna unguiculata*)

With cowpea cv. IT10K-817-3 as the host plant, the percent relative symbiotic effectiveness in 2021 differed significantly among the isolates, (*p* < 0.05) with values ranging from 16 to 97% (Table 1). Isolates were, respectively, classified as highly effective (≥80%), moderately effective (50–80%), and lowly effective (≤50%). Based on that, isolates TUTGmGH2 (37.83%), TUTGmGH3 (37.34%), TUTVsES1 (16.45%) and TUTVsES2 (29.50%) were ineffective. Isolate TUTVuSA3 (67.10%) was moderately effective, while isolates TUTVuSA1 (88.77%), TUTVuSA2 (91.38%) and the commercial strain (97.39%) were highly effective. However, with cowpea cv. IT10K-866-1 as the host plant, the relative symbiotic effectiveness values were much higher and ranged from 17.70 to 161.73%. Isolates TUTGmGH3, TUTGmGH2 and TUTVsES1 showed low effectiveness (17.70 to 35.80%). Isolate TUTPvES3 was moderately effective (60.49%), while four isolates and the commercial strain were highly effective with RSE values of 83.5%, 111.1%, 120.6%, 148.2% and 161.7% for TUTGmGH1, the inoculant strain, TUTVuSA3, TUTVuSA2 and TUTVuSA1, respectively.

#### 3.5.2. Bambara Groundnut (*Vigna subterranea*)

With Bambara groundnut landrace SSD5 as the host plant in 2021, only one isolate TUTVsES1 and the commercial strain were highly effective, with RSE values of 85.1% and 95.0%, respectively (Table 1). With Bambara groundnut landrace SSD8 as the host plant in 2022, only isolate TUTMgSA3 and TUTPvES2 were ineffective. The remaining six isolates plus the inoculant strain were highly effective (Table 2).

#### 3.5.3. Kersting’s Groundnut (*Macrotyloma geocarpum*)

With Kersting’s groundnut landrace Puffeun, TUTGmGH1, TUTPvES1 and TUTPvES3 were ineffective, and TUTMgSA1 and TUTVuSA3 were moderately effective, while TUTMgSA2, TUTMgSA3 and the inoculant strain were highly effective (Table 1). With landrace Dowie as the host plant, only one isolate TUTPvES3 (48.33%) was ineffective. Three other isolates TUTVuSA3 (53.89%), TUTPvES1 (55.56%) and TUTGmGH2 (79.44%) were moderately effective, while isolates TUTMgSA1 (177.78%), TUTMgSA2 (162.78%), TUTMgSA3 (116.67%), TUTGmGH3 (83.33%) and the commercial strain (94.44%) were highly effective (Table 2).

#### 3.5.4. Common Bean (*Phaseolus vulgaris*)

With common bean cv. NUA 734 as the host plant, none of the isolates were ineffective. Here, isolates TUTGmGH1 (75.23%) and TUTGmGH2 (70.28%) were moderately effective, while isolates TUTPvES1, TUTPvES2, TUTPvES3 and the commercial inoculant strain were highly effective, with RSE value ranging from 85.76 to 145.51% (Table 1). With the common bean cv. NUA 721 as the host plant, all the isolates were highly effective, with RSE values ranging from 94.17 to 147.99% (Table 2).

#### 3.5.5. Soybean (*Glycine max*)

When the soybean cv. TGX1740-2F was used as the host plant in 2021, only one isolate TUTPvES3 (67.88%) was moderately effective (Table 1). The other isolates were highly effective, with RSE values ranging from 82.48 to 233.58% (Table 1). With the common bean cv. TGX1937-1F as the host plant in 2022, isolate TUTPvES3 (44.93%) showed ineffective nodulation (Table 2). Three other isolates, TUTMgSA3 (59.42%), TUTVsES2 (69.08%) and TUTPvES2 (66.18%), were moderately effective, while four isolates, TUTGmGH1 (119.32%), TUTGmGH2 (135.27%), TUTGmGH3 (100.00%), TUTVuSA1 (95.17%) and the commercial inoculant strain (93.24%) were highly effective (Table 2).

#### 3.5.6. Velvet Bean (*Mucuna pruriens*)

With velvet cv. IIHR PS2 as the host in 2022, isolates TUTVuSA2 (75.21%), TUTVsES1 (79.89%), TUTVsES2 (60.61%) and TUTPvES2 (57.02%) were moderately effective, while the other isolates were highly effective, with RSE values ranging from 81.82% for TUTMgSA2 to 110.19% for TUTVuSA3 (Table 2).

#### 3.5.7. Jack Bean (*Canavalia ensiformis*)

With the jack bean Accession 498 as the host plant in 2022, all the isolates were highly effective, with RSE values ranging from 102.12% for TUTVuSA2 to 135.21% for TUTVsES1 (Table 2).

#### 3.5.8. Pigeonpea (*Cajanus cajan*)

When the pigeonpea cv. ICEAP00850 was used as host plant in 2022, isolates TUTVuSA1 (49.70%), TUTVsES1 (37.72%) and TUTMgSA1 (46.11%) were ineffective; isolates TUTGmGH2 (67.66%) and TUTGmGH3 (76.05%) were moderately effective, while isolate TUTPvES2 (115.57%) was highly effective (Table 2).

#### 3.5.9. Mungbean (*Vigna radiata*)

With the mungbean cv. VC6153 (B-20P) as the host plant in 2022, all the isolates were highly effective, with RSE values ranging from 100.00% (TUTMgSA2) to 172.73% (TUTGmGH2 and TUTGmGH3) (Table 2).

## 4. Discussion

Legume root nodulation by rhizobia is dependent on the compatibility between the host plant and microsymbiont. This compatibility is, in turn, dependent on mutual recognition of signals released by the two partners. Briefly, the legume would release flavonoid molecules (flavones, isoflavones, chalcones, etc.) that act as chemo attractants and nodulation (nod) gene-inducing signal compounds [24]. The compatible bacterial strain upon perceiving the plant signals would then release a lipo-chito-oligosaccharide nodulation signal (Nod factor), leading to root hair infection and nodule formation [25]. Legume species can however differ in the profile of the nod-gene-inducing signal molecules they release during courtship with the microsymbiont’s partner [26]. Conversely, the bacterial species also differ in the profile of the nod factors they synthesize and release in response to the host plant [27]. The signals released by both the legume plant and rhizobial bacterium form the basis for nodulation specificity between legumes and rhizobia. Thus, pea rhizobia would generally nodulate pea but not soybean, and vice versa. In this study, three rhizobial strains originally isolated from cowpea, Kersting’s groundnut, Bambara groundnut, common bean and soybean were used to inoculate 11 legume species in a cross-infectivity assay.

### 4.1. Nodulation and Shoot Dry Matter Accumulation Induced by Rhizobial Isolates

This study assessed nodulation and shoot dry matter (shoot DM) accumulation by different legume species inoculated by diverse rhizobial isolates under glasshouse conditions in 2021 and 2022. For this, fifteen (15) rhizobial isolates obtained from the root nodules of different legume species were used in cross-infectivity tests involving cowpea, Bambara groundnut, Kersting’s groundnut, common bean, soybean, winged bean, velvet bean, pigeonpea, jack bean, mungbean and chickpea as host plants. As to be expected, all the test isolates nodulated their homologous legume hosts from which they were originally isolated; however, the commercial inoculant strain used as a positive control was found to nodulate only cowpea, Bambara groundnut, Kersting’s groundnut, common bean, and soybean but not winged bean, velvet bean, jack bean, pigeonpea and mungbean. However, some test isolates exhibited some level of promiscuity; for example, isolate TUTGmGH3, which was originally isolated from the root nodule of soybean in Ghana, exhibited a high level of promiscuity as it nodulated eight host legume species in 2021 and seven in 2022. Similarly, the soybean isolate TUTGmGH2 nodulated seven species in addition to its homologous host in 2021 and also nodulated six other legume species in a 2022 experiment (Figure 1A,B). Conversely, isolate TUTMgSA1 which was originally obtained from the root nodule of Kersting’s groundnut in Ghana was the least promiscuous; thus, aside from its original host, this isolate only nodulated velvet bean in 2021, as well as velvet bean and pigeonpea in 2022. However, the fact that soybean isolates effectively nodulated a wide range of host species (e.g., cowpea, Bambara groundnut, pigeonpea and velvet bean) was not surprising; an earlier work by Gyogluu et al. [28] had shown that the rhizobial symbionts of soybean can effectively nodulate several legume hosts. In this study, the test isolates failed to nodulate chickpea; this could be because the main rhizobial symbionts of chickpea belong to the genus *Mesorhizobium* [29]. Thus, the findings of this study demonstrate that rhizobia can vary in their level of promiscuity, with some strains nodulating only a limited number of hosts while others exhibit a much broader host range [30,31]. The commercial inoculant strain used in this study elicited a high number of nodules when compared to the other test isolates, indicating its broader compatibility with most of the host legume species (Figure 2A,B and Figure 3A,B). Furthermore, this study observed marked differences in plant growth promotion by the test isolates; it is worth noting that legumes that were inoculated with their original rhizobial isolates exhibited better growth which translated into higher shoot DM, followed by the commercial inoculant strain, and then the nitrate-fed plants.

### 4.2. N_2_ Fixation and %Relative Symbiotic Effectiveness of Rhizobial Isolates

This study further assessed symbiotic performance of different rhizobial isolates nodulating their original host legumes versus other legume species grown in the glasshouse. For each legume species, two cultivars or landraces were used (except for winged bean for which one cultivar was used) for the experiment in 2021 and repeated in 2022. The findings revealed a marked variation in the symbiotic performance of the isolates that nodulated the various legume species (Table 1 and Table 2). There was a general tendency for these test rhizobial isolates to elicit better plant growth in their original host legumes; however, the symbiotic performance of these isolates in symbiosis with their original host were largely matched by the commercial inoculant strain. Aside from these general trends, however, there were instances where rhizobial isolates such as TUTGmGH1, TUTGmGH2 and TUTGmGH3 obtained from the root nodules of soybean elicited better symbiotic performance across diverse legume hosts. Moreover, in most instances, greater shoot dry matter accumulation resulting from inoculation was associated with increased shoot N concentration and N-fixed (Table 1 and Table 2). With cowpea for example, the plants inoculated with isolates TUTVuSA1, TUTVuSA2, TUTVuSA3, TUTGmGH2 and TUTGmGH3 as well as the commercial *Bradyrhizobium* strain (CB756) revealed lower δ^15^N values, higher N concentrations and amounts of N-fixed in both host plant genotypes tested. The N-fixed values for cowpea cv. IT10K-817-3 inoculated with the different isolates ranged from 5.68 to 128.21 mg.plant^−1^ and from 6.57 to 90.43 mg.plant^−1^ for cv. IT10K-866-1. The observed wide variability in the range of N-fixed by the different legume/rhizobia combinations suggests a complex pattern of compatibility between symbionts. Thus, the success of rhizobial inoculation is markedly influenced by compatibility between rhizobial strain and the legume genotype; this is not surprising, given that the legume/rhizobia symbiosis is known to exhibit some level of host–symbiont specificity [32]. Even when the same legume species was used, the choice of host genotype was found to influence symbiotic performance.

Comparing the symbiotic effectiveness of inoculant strains for each legume species with the test isolates revealed an interesting trend. In the common bean, for example, isolate TUTPvES1 elicited a greater %RSE than the commercial inoculant strain in 2021; however, the reverse trend was observed in 2022, indicating that symbiotic efficacy can be influenced by external environmental factors. However, for cowpea, Bambara groundnut and Kersting’s bean, the inoculant strain CB756 recorded the highest %RSE, indicating that the test isolates were symbiotically inferior to the inoculant strain in 2021, except for isolate TUTMgSA2 which recorded the same %RSE as the commercial inoculant containing *Bradyrhizobium* sp. strain CB756 (Table 1). With soybean, isolate TUTGmGH1 was symbiotically superior to the commercial inoculant strain in 2021. In 2022, however, the test rhizobial isolates were symbiotically superior to the inoculant strains of cowpea, Bambara groundnut, Kersting’s groundnut, soybean, velvet bean, jack bean, pigeonpea and mungbean (Table 2). It was only in common bean that the commercial inoculant strain outperformed all the test isolates in terms of symbiotic performance (Table 2). These findings suggest that, based on %RSE, most of the isolates tested in 2022 have potential for use in inoculant formation. In conclusion, the rhizobial isolates differed significantly in symbiotic performance and their ability to induce growth in host plants. The isolates also differed significantly in relative symbiotic effectiveness, offering opportunity for strain selection to improve the efficiency of the legume/rhizobium symbiosis.

## 5. Conclusions

Taken together, the native rhizobial isolates from root nodules of cowpea, Bambara groundnut, Kersting’s groundnut, common bean and soybean differed significantly in their ability to induce nodulation and shoot DM accumulation in different host plants. Further studies are recommended to explore these selected potential isolates in the field.

## Figures and Tables

**Table 1:** Symbiotic performance of diverse legume species inoculated with different rhizobial isolates under glasshouse conditions in 2021. Mean values (means ± se) with dissimilar letters in a column are significantly different at *p* ≤ 0.001 (***); *p* ≤ 0.01 (**); *p* ≤ 0.05 (*), ns = non significant at *p* ≥ 0.05. ND = not determined.

Legumes	Treatments	Shoot DM	δ^15^N	%N	N-Fixed	RSE
		g.plant^−1^	‰	%	mg.plant^−1^	%
Cowpea cv. IT10K-817-3	TUTVuSA1	3.40 ± 0.20 a	2.58 ± 0.05 c	0.84 ± 0.14 d	22.72 ± 1.29 de	88.77 ± 0.33 c
	TUTVuSA2	3.50 ± 0.30 a	0.99 ± 0.15 ef	1.62 ± 0.23 cd	46.38 ± 3.50 c	91.38 ± 0.54 b
	TUTVuSA3	2.43 ± 0.09 b	0.36 ± 0.01 f	3.66 ± 0.55 a	128.21 ± 9.30 a	67.10 ± 0.77 d
	TUTGmGH2	0.43 ± 0.03 d	4.96 ± 0.23 a	0.94 ± 0.14 d	20.37 ± 1.40 de	37.83 ± 0.54 e
	TUTGmGH3	1.43 ± 0.03 c	1.23 ± 0.03 e	2.69 ± 0.43 b	31.85 ± 1.82 d	37.34 ± 0.11 e
	TUTVsES1	0.63 ± 0.09 d	3.72 ± 0.56 b	0.94 ± 0.14 d	5.68 ± 0.65 f	16.45 ± 0.10 g
	TUTVsES2	1.13 ± 0.03 cd	2.03 ± 0.11 cd	1.23 ± 0.20 d	11.91 ± 0.36 ef	29.50 ± 0.21 f
	COMMERCIAL INOC	3.73 ± 0.49 a	1.32 ± 0.67 de	2.27 ± 0.32 bc	78.82 ± 6.05 b	97.39 ± 0.58 a
	NITRATE	3.83 ± 0.32 a	0.68 ± 0.34 ef	2.24 ± 0.33 bc	ND	ND
	F-STATISTIC	37.05 ***	38.52 ***	10.05 ***	108.73 ***	7.33 ***
Bambara groundnut LR.SSD5	TUTVsES1	3.43 ± 0.49 a	1.51 ± 0.43 a	2.99 ± 0.42 ab	126.89 ± 25.44 a	85.11 ± 0.31 b
	TUTVsES2	2.53 ± 0.28 bc	0.53 ± 0.07 b	1.85 ± 0.26 bc	43.53 ± 5.49 bc	62.78 ± 0.18 d
	TUTVsES3	3.13 ± 0.54 ab	0.28 ± 0.07 b	1.87 ± 0.28 bc	57.36 ± 0.43 b	77.67 ± 0.23 c
	TUTMgSA3	1.50 ± 0.10 cd	0.39 ± 0.04 b	2.33 ± 0.34 a–c	28.90 ± 1.97 b–d	37.22 ± 0.39 e
	TUTPvES2	0.77 ± 0.03 d	0.31 ± 0.11 b	1.89 ± 0.27 bc	12.16 ± 0.5 d	19.11 ± 0.56 g
	TUTGmGH1	1.13 ± 0.03 d	0.23 ± 0.05 b	1.66 ± 0.23 c	16.54 ± 0.85 cd	28.05 ± 0.66 f
	TUTGmGH2	1.13 ± 0.03 d	0.65 ± 0.09 b	1.35 ± 0.21 c	13.74 ± 0.52 cd	28.04 ± 0.88 f
	TUTGmGH3	1.43 ± 0.09 cd	0.18 ± 0.08 b	1.36 ± 0.26 c	15.22 ± 1.35 cd	35.48 ± 0.76 ef
	COMMERCIAL INOC	3.83 ± 0.58 a	0.46 ± 0.07 b	3.15 ± 0.78 a	107.51 ± 16.06 a	95.04 ± 0.54 a
	NITRATE	4.03 ± 1.27 a	0.43 ± 0.12 b	1.95 ± 0.28 bc	ND	ND
	F-STATISTIC	9.44 ***	5.85 ***	3.11 *	19.28 ***	5.98 ***
Kersting’s groundnut LR.Puffeun	TUTMgSA1	1.20 ± 0.75 c	0.96 ± 0.13 c	1.54 ± 0.22 bc	65.37 ± 1.71 c	54.55 ± 0.89 cd
	TUTMgSA2	1.97 ± 0.09 a	0.83 ± 0.04 cd	1.00 ± 0.15 c	39.00 ± 3.60 d	89.54 ± 0.88 a
	TUTMgSA3	1.83 ± 0.15 b	0.21 ± 0.01 g	2.94 ± 0.42 a	82.01 ± 1.53 b	83.18 ± 0.60 b
	TUTVuSA3	1.33 ± 0.15 c	0.48 ± 0.09 ef	1.29 ± 0.20 bc	33.11 ± 0.64 de	60.45 ± 0.98 c
	TUTPvES1	0.87 ± 0.09 d	0.23 ± 0.05 g	1.52 ± 0.21 bc	10.46 ± 0.46 fg	39.55 ± 0.78 f
	TUTPvES3	0.97 ± 0.07 d	0.29 ± 0.10 fg	1.87 ± 0.28 b	15.99 ± 1.18 f	44.10 ± 0.92 d
	TUTGmGH1	0.77 ± 0.07 d	1.26 ± 0.06 b	0.99 ± 0.14 c	5.95 ± 0.04 g	35.00 ± 0.67 g
	TUTGmGH3	1.00 ± 0.06 c	0.61 ± 0.03 de	0.98 ± 0.16 c	31.08 ± 1.71 e	45.45 ± 0.87 e
	COMMERCIAL INOC	1.97 ± 0.78 a	0.64 ± 0.03 de	1.50 ± 0.21 bc	97.62 ± 5.72 a	89.55 ± 0.77 a
	NITRATE	2.20 ± 0.36 a	1.67 ± 0.15 a	0.92 ± 0.14 c	ND	ND
	F-STATISTIC	25.28 ***	31.01 ***	7.55 ***	204.73 **	6.59 ***
Common bean cv. NUA 734	TUTPvES1	4.70 ± 2.60 a	3.80 ± 0.12 b	2.93 ± 0.43 a	50.72 ± 12.59 cd	145.51 ± 1.00 a
	TUTPvES2	3.33 ± 0.12 c	3.98 ± 0.02 b	2.23 ± 0.33 ab	65.77 ± 2.24 c	103.10. ± 0.88 c
	TUTPvES3	2.77 ± 0.18 d	2.30 ± 0.02 c	3.01 ± 0.43 a	90.21 ± 8.05 b	85.76 ± 0.81 d
	TUTGmGH2	2.43 ± 0.12 d	3.51 ± 0.06 b	2.48 ± 0.34 ab	51.14 ± 0.95 cd	75.23 ± 0.79 e
	TUTGmGH3	2.27 ± 0.07 d	4.06 ± 0.52 a	1.56 ± 0.22 b	46.19 ± 0.56 c–e	70.28 ± 0.74 ef
	COMMERCIAL INOC	4.30 ± 0.96 b	4.08 ± 0.00 a	2.93 ± 0.43 a	171.87 ± 15.81 a	133.13 ± 0.93 b
	NITRATE	3.23 ± 0.44 c	3.97 ± 0.10 b	1.24 ± 0.20 b	ND	ND
	F-STATISTIC	11.01 ***	15.19 ***	3.15 *	33.24 ***	7.57 ***
Soybean cv. TGX1740-2F	TUTGmGH1	3.20 ± 0.78 a	1.50 ± 0.00 b–d	3.69 ± 0.54 a	52.14 ± 3.78 cd	233.58 ± 3.66 a
	TUTGmGH2	2.47 ± 0.57 a	0.23 ± 0.10 g	2.72 ± 0.50 a–c	119.32 ± 19.94 a	180.29 ± 2.65 d
	TUTGmGH3	2.53 ± 1.39 a	0.27 ± 0.07 fg	2.15 ± 0.31 b–d	107.88 ± 25.30 ab	184.67 ± 3.06 c
	TUTVuSA1	1.27 ± 0.07 b	1.11 ± 0.04 c–e	3.17 ± 0.44 ab	37.60 ± 5.64 cd	92.70 ± 3.01 g
	TUTMgSA3	0.77 ± 0.07 b	3.11 ± 0.12 a	2.03 ± 0.29 b–e	18.28 ± 3.58 d	82.48 ± 1.77 h
	TUTVsES2	1.73 ± 0.42 b	2.11 ± 0.03 b	1.15 ± 0.16 de	15.17 ± 3.56 d	126.28 ± 1.98 e
	TUTPvES2	1.53 ± 0.07 b	0.06 ± 0.02 g	2.49 ± 0.36 bc	34.16 ± 0.05 cd	111.68 ± 1.82 f
	TUTPvES3	0.93 ± 0.15 b	3.48 ± 0.59 a	1.64 ± 0.30 c–e	13.68 ± 3.33 d	67.88 ± 1.07 i
	COMMERCIAL INOC	3.17 ± 2.32 a	0.89 ± 0.19 d–f	1.26 ± 0.18 de	73.01 ± 26.88 bc	231.39 ± 4.04 b
	NITRATE	1.37 ± 0.03 b	1.59 ± 0.21 bc	0.98 ± 0.23 e	ND	ND
	F-STATISTIC	7.44 ***	31.56 ***	5.99 ***	8.21 ***	6.45 ***
Winged bean cv.VRWB 4A	TUTMgSA2	1.43 ± 0.09 c	2.05 ± 0.18 a	1.72 ± 0.24 c	21.36 ± 0.70 e	ND
	TUTVsES1	2.03 ± 0.12 b	1.87 ± 0.06 a	2.42 ± 0.34 a–c	41.70 ± 2.98 c	ND
	TUTPvES3	1.90 ± 0.12 b	2.01 ± 0.09 a	2.26 ± 0.32 a–c	35.08 ± 1.80 d	ND
	TUTGmGH1	2.93 ± 0.17 a	1.86 ± 0.01 a	3.24 ± 0.47 ab	85.87 ± 0.72 a	ND
	TUTGmGH2	2.03 ± 0.12 b	0.51 ± 0.23 c	3.39 ± 0.54 a	56.90 ± 0.43 b	ND
	TUTGmGH3	1.77 ± 0.18 bc	1.30 ± 0.03 b	1.99 ± 0.28 bc	32.45 ± 1.76 d	ND
	F-STATISTIC	13.86 ***	21.78 ***	3.16 *	193.25 ***	
Velvet bean cv. IIHR PS 1	TUTVuSA1	2.67 ± 0.12 de	0.97 ± 0.04 ab	1.33 ± 0.19 a	31.35 ± 0.99 h	ND
	TUTVuSA2	4.43 ± 0.20 a	0.66 ± 0.13 b–e	1.21 ± 0.17 a	46.28 ± 2.10 b	ND
	TUTVuSA3	4.33 ± 0.23 a	0.18 ± 0.05 fg	1.36 ± 0.20 a	50.12 ± 2.81 a	ND
	TUTMgSA1	3.30 ± 0.15 bc	0.33 ± 0.05 e–g	1.27 ± 0.18 a	36.31 ± 1.28 fg	ND
	TUTMgSA2	3.43 ± 0.07 bc	0.77 ± 0.17	1.38 ± 0.20 a	40.31 ± 0.98 de	ND
	TUTMgSA3	2.67 ± 0.09 de	0.62 ± 0.02 b–e	1.17 ± 0.17 a	27.51 ± 0.48 i	ND
	TUTVsES1	3.10 ± 0.17 b–d	0.25 ± 0.08 e–g	1.38 ± 0.20 a	38.51 ± 0.93 d–f	ND
	TUTVsES3	3.03 ± 0.18 cd	1.06 ± 0.03 a	1.19 ± 0.17 a	32.79 ± 0.31	ND
	TUTPvES2	2.33 ± 0.19 d	0.86 ± 0.18 a–c	1.23 ± 0.18 a	22.58 ± 0.43 j	ND
	TUTPvES3	3.63 ± 0.15 b	0.60 ± 0.12 b–e	1.30 ± 0.19 a	40.64 ± 1.08 de	ND
	TUTGmGH1	4.23 ± 0.20 a	0.49 ± 0.28 c–f	1.22 ± 0.17 a	44.68 ± 1.03 bc	ND
	TUTGmGH2	3.60 ± 0.17 b	0.07 ± 0.01 g	1.32 ± 0.19 a	42.38 ± 0.98 cd	ND
	TUTGmGH3	3.50 ± 0.12 bc	0.38 ± 0.10 d–g	1.22 ± 0.17 a	37.78 ± 0.19 ef	ND
	F-STATISTIC	16.21 ***	6.37 ***	0.16 ns	37.74 ***	
Jack bean cv. Accession 493	TUTVuSA2	4.60 ± 0.29 a	1.13 ± 0.06 d	1.25 ± 0.21 a	45.58 ± 1.62 a	ND
	TUTMgSA3	2.80 ± 0.10 de	3.07 ± 0.04 b	1.44 ± 0.21 a	33.83 ± 1.07 b	ND
	TUTVsES1	2.53 ± 0.09 e	3.38 ± 0.08 a	1.58 ± 00.22 a	35.37 ± 0.94 b	ND
	TUTVsES2	3.53 ± 0.09 b	2.82 ± 0.08 c	1.51 ± 0.22 a	45.81 ± 1.32 a	ND
	TUTPvES2	2.00 ± 0.10 f	3.32 ± 0.06 a	1.59 ± 0.27 a	28.56 ± 0.12 c	ND
	TUTVsES3	3.23 ± 0.09 bc	2.83 ± 0.03 c	1.62 ± 0.22 a	44.08 ± 0.32 a	ND
	F-STATISTIC	38.61 ***	193.69 ***	0.40 ns	48.87 ***	
Pigeonpea cv. ICEAP500557	TUTVuSA1	0.87 ± 0.03 b	0.76 ± 0.08 c	1.12 ± 0.16 b	8.82 ± 0.83 c	ND
	TUTVuSA2	0.97 ± 0.09 ab	0.76 ± 0.22 c	1.19 ± 0.23 b	9.11 ± 1.61 c	ND
	TUTMgSA1	0.77 ± 0.09 b	3.03 ± 0.12 a	1.32 ± 0.19 b	9.85 ± 1.02 bc	ND
	TUTGmGH1	0.97 ± 0.09 ab	1.36 ± 0.03 b	1.26 ± 0.19 b	11.26 ± 0.02 bc	ND
	TUTGmGH2	1.17 ± 0.07 a	0.16 ± 0.04 d	1.53 ± 0.21 b	15.99 ± 1.42 a	ND
	TUTGmGH3	0.70 ± 0.10 b	1.20 ± 0.07 b	2.47 ± 0.35 a	12.75 ± 0.33 b	ND
	F-STATISTIC	4.28 *	74.99 ***	4.81 *	6.91 **	
Mungbean cv. VC1973A	TUTMgSA2	0.90 ± 0.12 b	1.45 ± 0.28 c	1.49 ± 0.23 b	10.09 ± 0.41 b	ND
	TUTMgSA3	0.90 ± 0.00 b	2.07 ± 0.02 b	1.26 ± 0.20 b	9.59 ± 0.49 b	ND
	TUTPvES1	0.77 ± 0.09 b	2.29 ± 0.29 b	1.52 ± 0.22 b	11.08 ± 0.25 b	ND
	TUTGmGH1	0.87 ± 0.09 b	4.16 ± 0.13 a	1.42 ± 0.21 b	9.59 ± 0.44 b	ND
	TUTGmGH3	1.17 ± 0.03 a	1.28 ± 0.04 c	3.36 ± 0.51 a	34.26 ± 1.00 a	ND
	F-STATISTIC	3.67 *	36.52 ***	8.52 **	348.88 ***	

**Table 2:** Symbiotic performance of diverse legume species inoculated with different rhizobial isolates under glasshouse conditions in 2022. Mean values (means ± se) with dissimilar letters in a column are significantly different at at *p* ≤ 0.001 (***); *p* ≤ 0.01 (**); *p* ≤ 0.05 (*), ns = non significant at *p* ≥ 0.05; ND = not determined.

Legumes	Treatments	Shoot DM	δ^15^N	N	N-Fixed	RSE
		g.plant^−1^	‰	%	mg.plant^−1^	%
Cowpea cv. IT10K-866-1	TUTVuSA1	3.93 ± 0.68 a	2.58 ± 0.05 c	0.71 ± 0.04 h	39.62 ± 1.44 c	161.73 ± 1.93 a
	TUTVuSA2	3.60 ± 0.21 b	3.63 ± 0.08 b	0.98 ± 0.06 fg	32.74 ± 4.17 d	148.15 ± 1.45 b
	TUTVuSA3	2.93 ± 0.43 c	1.26 ± 0.04 d	2.02 ± 0.01 d	49.46 ± 1.47 b	120.58 ± 1.63 c
	TUTGmGH1	2.03 ± 0.07 de	1.10 ± 0.04 d	2.56 ± 0.03 b	51.15 ± 1.99 b	83.54 ± 1.22 e
	TUTGmGH2	0.63 ± 0.09 h	0.38 ± 0.20 e	1.99 ± 0.08 d	12.74 ± 1.24 f	25.93 ± 1.07 h
	TUTGmGH3	0.43 ± 0.07 h	1.80 ± 0.15 cd	2.20 ± 0.03 c	8.86 ± 1.41 f	17.70 ± 1.42 i
	TUTVsES1	0.87 ± 0.03 gh	4.96 ± 0.24 a	0.82 ± 0.08 gh	27.97 ± 1.41 d	35.80 ± 0.69 g
	TUTPvES3	1.10 ± 0.10 fh	2.20 ± 0.11 c	1.34 ± 0.08 e	20.76 ± 1.59 e	60.49 ± 1.48 f
	COMMERCIAL INOC	2.70 ± 0.12 cd	0.34 ± 0.00 e	3.18 ± 0.01 a	90.43 ± 4.17 a	111.10 ± 1.51 d
	NITRATE	2.43 ± 0.12 cd	2.08 ± 0.71 c	0.84 ± 0.10 gh	ND	ND
	F-STATISTIC	71.80 ***	31.66 ***	216.17 **	135.81 **	6.98 ***
Bambara groundnut LR. SSD8	TUTVsES1	3.83 ± 0.43 a	0.62 ± 0.03 ab	2.26 ± 0.34 ab	73.25 ± 6.31 a	126.40 ± 2.65 b
	TUTVsES2	3.97 ± 0.13 a	0.63 ± 0.13 ab	2.06 ± 0.31 b	71.75 ± 2.13 a	131.02 ± 2.23 a
	TUTVsES3	2.50 ± 0.31 c	0.69 ± 0.13 a	2.95 ± 0.42 a	69.71 ± 9.81 a	82.51 ± 1.45 de
	TUTMgSA3	0.83 ± 0.03 e	0.23 ± 0.05 c	1.88 ± 0.34 b	13.21 ± 0.87 de	27.39 ± 1.32 e
	TUTPvES2	0.73 ± 0.03 e	0.65 ± 0.23 ab	1.29 ± 0.21 b	8.12 ± 0.16 e	24.09 ± 1.21 f
	TUTGmGH1	3.07 ± 0.09 bc	0.20 ± 0.09 c	1.41 ± 0.19 b	38.31 ± 1.71 b	101.32 ± 1.49 c
	TUTGmGH2	2.53 ± 0.32 c	0.69 ± 0.04 b	1.36 ± 0.20 b	24.10 ± 1.91 cd	83.50 ± 2.12 de
	TUTGmGH3	2.63 ± 0.07 bc	0.29 ± 0.04 bc	1.84 ± 0.25 b	42.93 ± 1.09 b	86.80 ± 1.97 d
	COMMERCIAL INOC	3.87 ± 0.09 a	0.40 ± 0.01 a–c	1.89 ± 0.27 b	63.78 ± 0.10 a	127.72 ± 2.22 b
	NITRATE	3.03 ± 0.47 bc	0.32 ± 0.17 a–c	1.77 ± 0.31 b	ND	ND
	F-STATISTIC	28.93 ***	3.07 *	2.79 *	42.04 ***	7.47 ***
Kersting’s groundnut LR. Dowie	TUTMgSA1	3.20 ± 0.10 a	0.88 ± 0.12 c	1.51 ± 0.22 b–d	48.09 ± 6.08 a	177.78 ± 4.56 a
	TUTMgSA2	2.93 ± 0.15 a	0.77 ± 0.32 c	1.07 ± 0.15 d	31.13 ± 3.17 b	162.78 ± 3.76 b
	TUTMgSA3	2.10 ± 0.17 b	0.43 ± 0.07 c	2.97 ± 0.42 a	54.41 ± 11.46 a	116.67 ± 3.81 c
	TUTVuSA3	0.97 ± 0.09 h–j	0.58 ± 0.10 c	1.14 ± 0.16 cd	10.80 ± 0.62 c	53.89 ± 3.42 f
	TUTPvES1	1.00 ± 0.06 h–j	0.37 ± 0.13 c	1.87 ± 0.28 bc	18.73 ± 2.98 bc	55.56 ± 2.77 f
	TUTPvES3	0.87 ± 0.09 h–j	0.38 ± 0.07 c	1.99 ± 0.30 b	17.70 ± 4.28 bc	48.33 ± 2.81 g
	TUTGmGH2	1.43 ± 0.15 d–f	2.46 ± 0.37 a	0.95 ± 0.13 d	13.94 ± 3.32 c	79.44 ± 2.12 ef
	TUTGmGH3	1.50 ± 0.10 c–e	0.28 ± 0.07 c	1.52 ± 0.21 b–d	22.71 ± 2.81 bc	83.33 ± 2.65 e
	COMMERCIAL INOC	1.70 ± 0.10 cd	1.27 ± 0.28 bc	1.12 ± 0.16 cd	18.86 ± 2.18 bc	94.44 ± 1.97 d
	NITRATE	1.80 ± 0.15 c	2.07 ± 0.79 ab	0.85 ± 0.12 d	ND	ND
	F-STATISTIC	49.86 ***	5.73 ***	7.06 ***	9.67 ***	8.01 ***
Common bean cv. NUA 721	TUTPvES1	3.10 ± 0.12 ab	4.31 ± 0.66 ab	2.78 ± 0.40 ab	85.37 ± 9.28 ab	139.01 ± 1.77 b
	TUTPvES2	2.80 ± 0.17 bc	1.13 ± 0.22 c	2.47 ± 0.35 a–c	70.14 ± 14.22 bc	125.56 ± 1.87 d
	TUTPvES3	3.03 ± 0.12 ab	2.82 ± 0.42 bc	3.22 ± 0.46 ab	69.53 ± 6.02 bc	135.87 ± 1.32 c
	TUTGmGH2	2.20 ± 0.21 de	4.98 ± 0.70 a	1.51 ± 0.21 cd	35.33 ± 9.22 d	98.65 ± 1.90 e
	TUTGmGH3	2.10 ± 0.15 df	5.64 ± 0.78 a	2.12 ± 0.30 bc	64.86 ± 11.34 bc	94.17 ± 1.66 ef
	COMMERCIAL INOC	3.30 ± 0.21 a	5.10 ± 0.73 a	3.02 ± 0.42 ab	98.03 ± 6.90 a	147.99 ± 1.84 a
	NITRATE	2.23 ± 0.09 de	4.68 ± 0.65 ab	0.86 ± 0.15 d	ND	ND
	F-STATISTIC	20.62 ***	6.29 **	6.01 **	9.55 ***	5.78 **
Soybean cv. TGX1937-1F	TUTGmGH1	2.47 ± 0.15 ab	1.58 ± 0.22 bc	3.82 ± 0.55 a	77.48 ± 7.82 a	119.32 ± 1.41 b
	TUTGmGH2	2.80 ± 0.15 a	0.43 ± 0.19 de	2.39 ± 0.34 b–e	67.66 ± 12.49 a	135.27 ± 1.54 a
	TUTGmGH3	2.07 ± 0.22 bc	0.39 ± 0.06 de	2.04 ± 0.28 d–f	43.13 ± 9.78 bc	100.00 ± 1.11 c
	TUTVuSA1	1.97 ± 0.09 c	1.43 ± 0.20 bc	3.55 ± 0.52 ab	68.98 ± 6.93 a	95.17 ± 1.45 d
	TUTMgSA3	1.23 ± 0.09 d–f	3.79 ± 0.56 a	1.20 ± 0.17 ef	14.56 ± 1.18 d	59.42 ± 0.87 f
	TUTVsES2	1.43 ± 0.09 d	2.05 ± 0.29 b	1.19 ± 0.17 ef	17.36 ± 3.56 d	69.08 ± 0.27 e
	TUTPvES2	1.37 ± 0.09 de	1.09 ± 0.15 cd	3.31 ± 0.50 a–c	45.80 ± 9.30 b	66.18 ± 1.42 ef
	TUTPvES3	0.93 ± 0.15 ef	0.15 ± 0.06 e	2.81 ± 0.46 a–d	26.47 ± 5.77 b–d	44.93 ± 0.77 g
	COMMERCIAL INOC	1.93 ± 0.09 c	1.16 ± 0.18 cd	1.12 ± 0.16 f	21.86 ± 4.20 cd	93.24 ± 0.56 de
	NITRATE	2.07 ± 0.18 bc	1.99 ± 0.29 b	1.14 ± 0.18 f	ND	ND
	F-STATISTIC	16.72 ***	17.53 ***	7.43 ***	11.19 ***	8.22 ***
Velvet bean cv. IIHR PS 2	TUTVuSA1	3.77 ± 0.20 ab	0.61 ± 0.11 ab	1.29 ± 0.18 a	47.81 ± 4.34 ab	103.86 ± 3.11 b
	TUTVuSA2	2.73 ± 0.09 e	0.51 ± 0.07 a–d	1.15 ± 0.18 a	31.16 ± 4.35 b–d	75.21 ± 2.02 f
	TUTVuSA3	4.00 ± 0.21 a	0.83 ± 0.12 a	1.29 ± 0.19 a	52.34 ± 10.37 a	110.19 ± 2.76 a
	TUTMgSA1	3.37 ± 0.12 b–d	0.18 ± 0.05 cd	1.23 ± 0.18 a	41.10 ± 4.77 a–c	92.84 ± 1.79 c
	TUTMgSA2	2.97 ± 0.29 c–e	0.52 ± 0.09 a–d	1.30 ± 0.19 a	39.70 ± 9.69 a–c	81.82 ± 1.82 d
	TUTMgSA3	3.40 ± 0.17 b–d	0.70 ± 0.10 ab	1.26 ± 0.18 a	42.89 ± 6.39 a–c	93.66 ± 1.54 c
	TUTVsES1	2.90 ± 0.12 de	0.15 ± 0.06 d	1.41 ± 0.20 a	40.58 ± 4.34 a–c	79.89 ± 1.44 e
	TUTVsES2	2.20 ± 0.20 f	0.69 ± 0.10 ab	1.54 ± 0.21 a	55.84 ± 7.90 a	60.61 ± 0.86 g
	TUTPvES2	3.43 ± 0.07 bc	0.90 ± 0.22 a	1.20 ± 0.20 a	24.90 ± 4.87 cd	57.02 ± 0.65 h
	TUTPvES3	3.60 ± 0.17 ab	0.35 ± 0.06 b–d	1.35 ± 0.19 a	46.59 ± 7.17 ab	94.49 ± 1.89 c
	NITRATE	3.63 ± 0.15 ab	0.55 ± 0.20 a–c	1.11 ± 0.16 a	ND	ND
	F-STATISTIC	22.32 ***	3.99 **	0.39 ns	3.68 **	6.06 **
Jack bean cv. Accession 498	TUTVuSA2	3.37 ± 0.09 e	0.32 ± 0.15 b	1.23 ± 0.17 b	41.33 ± 6.04 b	102.12 ± 3.23 f
	TUTMgSA3	4.33 ± 0.15 bc	3.43 ± 0.48 a	1.61 ± 0.23 b	69.35 ± 7.78 b	131.21 ± 3.44 b
	TUTVsES1	4.47 ± 0.18 b	3.70 ± 0.54 a	1.60 ± 0.23 b	72.41 ± 13.54 b	135.45 ± 2.67 a
	TUTVsES2	3.67 ± 0.09 de	3.85 ± 0.53 a	1.65 ± 0.24 b	59.98 ± 7.11 b	111.21 ± 3.02 e
	TUTVsES3	3.93 ± 0.19 cd	3.85 ± 0.53 b	1.65 ± 0.24 b	80.67 ± 13.74 b	119.09 ± 2.17 d
	TUTGmGH3	4.10 ± 0.00 bc	1.27 ± 0.200 b	3.43 ± 0.54 a	140.77 ± 22.08 a	124.24 ± 1.12 c
	NITRATE	4.87 ± 0.15 a	3.43 ± 0.49 b	1.61 ± 0.23 b	ND	ND
	F-STATISTIC	17.97 ***	8.95 ***	5.79 **	6.59 ***	8.88 **
Pigeonpea cv. ICEAP00850	TUTVuSA1	0.83 ± 0.07 c	1.11 ± 0.15 cd	2.04 ± 0.29 ab	17.15 ± 3.30 a	49.70 ± 0.35 d
	TUTVsES1	1.27 ± 0.07 b	2.31 ± 0.34 b	1.22 ± 0.21 bc	15.28 ± 2.06 a	37.72 ± 0.76 e
	TUTMgSA1	1.20 ± 0.06 b	3.64 ± 0.51 a	1.53 ± 0.21 a–c	18.49 ± 3.31 a	46.11 ± 1.23 d
	TUTPvES2	1.27 ± 0.03 b	1.06 ± 0.18 d	1.69 ± 0.24 a–c	21.29 ± 2.44 a	115.57 ± 1.09 a
	TUTGmGH2	1.27 ± 0.17 b	2.09 ± 0.29 bc	1.64 ± 0.23 a–c	21.47 ± 5.94 a	67.66 ± 0.91 c
	TUTGmGH3	0.97 ± 0.09 bc	1.61 ± 0.24 b–d	2.34 ± 0.35 a	22.04 ± 1.14 a	76.05 ± 0.29 b
	NITRATE	1.67 ± 0.12 a	1.53 ± 0.29 b–d	0.98 ± 0.15 c	ND	ND
	F-STATISTIC	9.16 ***	7.42 ***	3.06 *	0.86 ns	8.66 **
Mungbean cv. VC6 153 (B-20P)	TUTMgSA2	0.77 ± 0.03 c	2.78 ± 0.45 b	1.34 ± 0.19 cd	10.30 ± 1.61 c	100.00 ± 0.33 d
	TUTMgSA3	0.90 ± 0.00 c	1.53 ± 0.21 bc	2.25 ± 0.32 ab	21.50 ± 2.50 b	116.88 ± 0.87 c
	TUTPvES1	1.17 ± 0.12 ab	2.75 ± 0.55 b	1.52 ± 0.22 b–d	17.43 ± 1.70 bc	151.95 ± 0.76 b
	TUTGmGH2	1.33 ± 0.09 a	1.05 ± 0.30 c	1.55 ± 0.25 b–d	20.44 ± 2.72 bc	172.73 ± 0.94 a
	TUTGmGH3	1.33 ± 0.03 a	1.33 ± 0.20 c	2.77 ± 0.49 a	37.23 ± 7.47 a	172.73 ± 0.44 a
	NITRATE	0.97 ± 0.07 bc	4.40 ± 0.74 a	1.05 ± 0.17 d	ND	ND
	F-STATISTIC	11.53 ***	7.07 **	4.46 **	7.64 ***	

## Data Availability

The original contributions presented in this study are included in the article/supplementary material. Further inquiries can be directed to the corresponding author.

## Supplementary Materials

The following supporting information can be downloaded at: https://www.mdpi.com/article/10.3390/microorganisms13112463/s1, Table S1: Nodulation of diverse legume species by native rhizobial isolates planted under glasshouse conditions in 2021. X = non nodulated, ✓ = nodulated. Table S2: Nodulation of diverse legume species by native rhizobial isolates planted under glasshouse conditions in 2022. X = non nodulated, ✓ = nodulated, ND = not determined. Table S3: Nodule number and nodule dry matter of legume species used in cross-infectivity test in the glasshouse in 2021. Table S4: Nodule number and nodule dry matter of legume species used in cross-infectivity test in the glasshouse in 2022.

## Abbreviations

The following abbreviations are used in this manuscript:

%RSE	Percent relative symbiotic effectiveness
N_2_	Nitrogen
DM	Dry matter
