# Supplementary material for: Cross-Infectivity of 11 Different Legume Species by 15 Native Rhizobia Isolated from African Soils
Source: Microorganisms. 2025 Oct 28;13(11):2463. doi: 10.3390/microorganisms13112463 (PMC12654437; doi:10.3390/microorganisms13112463)
Supplement: Supplementary file 1 [file microorganisms-13-02463-s001.zip › microorganisms-3893257-supplementary.pdf]

Table S1: Nodulation of diverse legume species by native rhizobial isolates planted under glasshouse conditions in 2021. X=non nodulated, ✓=nodulated.

Isolate	**Cowpea [IT10K-817-3]**	**Kersting's groundnut [Puffeun]**	**Bambara groundnut [SSD5]**	**Common bean [NUA 734]**	**Soybean [TGx17402F]**	**Jack bean [493]**	**Winged bean [VRWB 4A]**	**Mung bean [VC1973A]**	**Pigeonpea [ICEAP500557]**	**Chickpea [Desi]**	**Velvet [IIHRPS5]**
**TUTVuSA1**	✓	X	X	X	✓	X	X	X	✓	X	✓
**TUTVuSA2**	✓	X	X	X	X	✓	X	✓	✓	X	✓
**TUTVuSA3**	✓	✓	X	X	X	X	X	X	X	X	✓
**TUTMgSA1**	X	✓	X	X	X	X	X	X	✓	X	✓
**TUTMgSA2**	X	✓	X	X	✓	X	✓	✓	X	X	✓
**TUTMgSA3**	X	✓	✓	X	✓	✓	X	✓	X	X	✓
**TUTVsES1**	X	X	✓	X	X	✓	✓	X	X	X	✓
**TUTVsES2**	X	X	✓	X	✓	✓	X	X	X	X	X
**TUTVsES3**	X	X	✓	X	X	✓	X	X	X	X	✓
**TUTPvES1**	X	✓	X	✓	X	X	X	✓	X	X	X
**TUTPvES2**	X	X	X	✓	✓	✓	X	X	✓	X	X
**TUTPvES3**	✓	✓	X	✓	✓	X	✓	X	X	X	✓
**TUTGmGH1**	✓	X	✓	X	✓	X	✓	X	✓	X	✓
**TUTGmGH2**	✓	✓	✓	✓	✓	X	X	✓	✓	X	✓
**TUTGmGH3**	✓	✓	✓	✓	✓	X	✓	✓	✓	X	✓

Table S2: Nodulation of diverse legume species by native rhizobial isolates planted under glasshouse conditions in 2022. X=non nodulated, ✓=nodulated, ND= not determined.

Isolate	**Cowpea [IT10K8661]**	**Kersting's groundnut [Dowie]**	**Bambara groundnut [SSD8]**	**Common bean [NUA 721]**	**Soybean [TGx19371F]**	**Jack bean [498]**	**Winged bean [N/A]**	**Mung bean [VC6153(B20P)]**	**Pigeonpea [ICEAP00850]**	**Chickpea [Cabuli]**	**Velvet [IIRPS2]**
**TUTVuSA1**	✓	X	X	X	✓	X	ND	X	✓	X	✓
**TUTVuSA2**	✓	X	X	X	X	✓	ND	X	X	X	✓
**TUTVuSA3**	✓	✓	X	X	X	X	ND	X	X	X	✓
**TUTMgSA1**	X	✓	X	X	X	X	ND	X	✓	X	✓
**TUTMgSA2**	X	✓	X	X	X	X	ND	✓	X	X	✓
**TUTMgSA3**	X	✓	✓	X	✓	✓	ND	✓	X	X	✓
**TUTVsES1**	✓	X	✓	X	X	✓	ND	X	✓	X	✓
**TUTVsES2**	✓	X	✓	X	✓	✓	ND	X	X	X	X
**TUTVsES3**	X	X	✓	X	X	✓	ND	X	X	X	✓
**TUTPvES1**	X	✓	X	✓	X	X	ND	✓	X	X	X
**TUTPvES2**	X	X	✓	✓	✓	X	ND	X	✓	X	✓
**TUTPvES3**	✓	✓	X	✓	✓	X	ND	X	X	X	✓
**TUTGmGH1**	✓	X	✓	X	✓	X	ND	X	X	X	X
**TUTGmGH2**	✓	✓	✓	✓	✓	X	ND	✓	✓	X	X
**TUTGmGH3**	✓	✓	✓	✓	✓	✓	ND	✓	✓	X	X

Table S3: Nodule number and nodule dry matter of legume species used in cross-infectivity test in the glasshouse in 2021.

Treatment	Cowpea cv. IT10K-817-3		Bambara groundnut LR.SSD5		Kersting's groundnut LR.Puffeun		Common bean cv. NUA 734		Soybean cv. TGX1740-2F	
	Nodule no. per plant	Nodule DM mg. plant $^{-1}$	Nodule no. per plant	Nodule DM mg. plant $^{-1}$	Nodule no. Per plant	Nodule DM mg. plant $^{-1}$	Nodule no. per plant	Nodule DM mg. plant $^{-1}$	Nodule no. per plant	Nodule DM mg. plant $^{-1}$
TUTVuSA1	2.33±0.67b	0.11±0.01d	0.0±0.0	0.0±0.0	0.0±0.0	0.0±0.0	0.0±0.0	0.0±0.0	19.67±2.33b	0.28±0.02cd
TUTVuSA2	46.67±19.06a	0.87±0.21a	0.0±0.0	0.0±0.0	0.0±0.0	0.0±0.0	0.0±0.0	0.0±0.0	0.0±0.0	0.0±0.0
TUTVuSA3	24.67±1.45ab	0.51±0.03bc	0.0±0.0	0.0±0.0	15.33±2.33e	0.17±0.04c	0.0±0.0	0.0±0.0	0.0±0.0	0.0±0.0
TUTMgSA1	0.0±0.0	0.0±0.0	0.0±0.0	0.0±0.0	97.33±14.38bc	1.11±0.22a	0.0±0.0	0.0±0.0	0.0±0.0	0.0±0.0
TUTMgSA2	0.0±0.0	0.0±0.0	0.0±0.0	0.0±0.0	38.67±2.03d	0.40±0.01bc	0.0±0.0	0.0±0.0	4.11±0.03c	0.21±0.01de
TUTMgSA3	0.0±0.0	0.0±0.0	7.0±1.00b	0.22±0.03d	21.67±1.45de	0.29±0.04bc	0.0±0.0	0.0±0.0	4.67±1.76c	0.21±0.08de
TUTVsES1	0.0±0.0	0.0±0.0	40.67±13.9c	0.52±0.11b	0.0±0.0	0.0±0.0	0.0±0.0	0.0±0.0	0.0±0.0	0.0±0.0
TUTVsES2	0.0±0.0	0.0±0.0	97.0±5.19a	1.12±0.21a	0.0±0.0	0.0±0.0	0.0±0.0	0.0±0.0	1.33±0.33c	0.12±0.00e
TUTVsES3	0.0±0.0	0.0±0.0	30.33±2.03b	0.39±0.01c	0.0±0.0	0.0±0.0	0.0±0.0	0.0±0.0	0.0±0.0	0.0±0.0
TUTPvES1	0.0±0.0	0.0±0.0	0.0±0.0	0.0±0.0	11.00±1.15d	0.14±0.00c	7.67±0.67ab	0.16±0.01bc	0.0±0.0	0.0±0.0
TUTPvES2	0.0±0.0	0.0±0.0	0.0±0.0	0.0±0.0	0.0±0.0	0.0±0.0	5.67±0.33b	0.21±0.01ab	2.00±0.58c	0.12±0.01e
TUTPvES3	2.11±0.59b	0.09±0.02d	0.0±0.0	0.0±0.0	27.33±5.24de	0.27±0.05bc	12.00±1.53a	0.27±0.03a	2.33±0.88c	0.13±0.01e
TUTGmGH1	2.67±0.67b	0.08±0.02d	13.0±0.58b	0.23±0.01d	0.0±0.0	0.0±0.0	0.0±0.0	0.0±0.0	42.00±3.21a	0.64±0.04a
TUTGmGH2	4.72±0.88b	0.0±0.0	7.0±3.06c	0.19±0.07e	61.00±3.79c	0.50±0.04b	3.67±0.88b	0.11±0.03c	6.33±0.88c	0.37±0.38bc
TUTGmGH3	19.67±2.03ab	0.24±0.01cd	15.33±0.67b	0.21±0.01d	109.33±3.76b	1.15±0.01a	7.25±3.09ab	0.15±0.03bc	18.00±1.73b	0.31±0.03cd
COMMERCIAL INOC	50.67±12.81a	0.61±0.15ab	101.0±26.7a	1.05±0.22a	149.67±6.69a	1.33±0.11a	8.33±1.20ab	0.20±0.02b	6.33±0.88c	0.47±0.07b
F-statistics	4.87*	8.66**	12.68***	10.92***	67.58***	28.63***	2.09ns	4.64***	64.62***	17.95***

Table S3: Continue

Treatment	Winged bean cv.VRWB 4A		Velvet bean cv. IIHR PS 1		Jack bean cv. Accession 493		Pigeonpea cv. ICEAP500557		Mungbean cv. VC1973A	
	Nodule no. per plant	Nodule DM mg. plant $^{-1}$	Nodule no. per plant	Nodule DM mg. plant $^{-1}$	Nodule no. per plant	Nodule DM mg. plant $^{-1}$	Nodule no. per plant	Nodule DM mg. plant $^{-1}$	Nodule no. per plant	Nodule DM mg. plant $^{-1}$
TUTVuSA1	0.0±0.0	0.0±0.0	4.33±0.88bc	0.09±0.02b	0.0±0.0	0.0±0.0	5.33±1.45e	0.13±0.01e	0.0±0.0	0.0±0.0
TUTVuSA2	0.0±0.0	0.0±0.0	3.33±0.88c	0.12±0.01b	2.67±0.88b	0.09±0.01c	2.67±0.67e	0.12±0.00e	3.03±0.0	0.09±0.01c
TUTVuSA3	0.0±0.0	0.0±0.0	6.33±1.76bc	0.16±0.02b	0.0±0.0	0.0±0.0	0.0±0.0	0.0±0.0	0.0±0.0	0.0±0.0
TUTMgSA1	0.0±0.0	0.0±0.0	3.33±0.33c	0.13±0.00b	0.0±0.0	0.0±0.0	29.33±2.33d	0.26±0.03d	0.0±0.0	0.0±0.0
TUTMgSA2	4.67±1.76b	0.12±0.03a	4.33±2.03bc	0.14±0.07b	0.0±0.0	0.0±0.0	0.0±0.0	0.0±0.0	1.67±0.67c	0.11±0.00bc
TUTMgSA3	0.0±0.0	0.0±0.0	84.33±8.41a	0.98±0.08a	1.67±0.67b	0.12±0.01ac	0.0±0.0	0.0±0.0	2.00±1.00c	0.12±0.01bc
TUTVsES1	8.33±2.60b	0.15±0.07a	13.67±2.33b	0.17±0.04b	2.67±0.88b	0.11±0.01bc	0.0±0.0	0.0±0.0	0.0±0.0	0.0±0.0
TUTVsES2	0.0±0.0	0.0±0.0	0.0±0.0	0.0±0.0	3.33±0.88b	0.13±0.01ab	0.0±0.0	0.0±0.0	0.0±0.0	0.0±0.0
TUTVsES3	0.0±0.0	0.0±0.0	5.00±1.00bc	0.11±0.02b	1.67±0.33b	0.10±0.01bc	0.0±0.0	0.0±0.0	0.0±0.0	0.0±0.0
TUTPvES1	0.0±0.0	0.0±0.0	0.0±0.0	0.0±0.0	0.0±0.0	0.0±0.0	0.0±0.0	0.0±0.0	6.33±1.20b	0.14±0.12b
TUTPvES2	0.0±0.0	0.0±0.0	4.67±0.88bc	0.14±0.03b	6.00±1.15a	0.15±0.01a	6.33±1.45e	0.16±0.01e	0.0±0.0	0.0±0.0
TUTPvES3	3.00±0.58b	0.12±0.03a	3.33±1.45c	0.09±0.04b	0.0±0.0	0.0±0.0	0.0±0.0	0.0±0.0	0.0±0.0	0.0±0.0
TUTGmGH1	5.33±1.86b	0.13±0.03a	9.00±2.65bc	0.14±0.01b	0.0±0.0	0.0±0.0	40.33±1.45c	0.36±0.02c	0.0±0.0	0.0±0.0
TUTGmGH2	5.00±1.15b	0.22±0.03a	3.67±1.33c	0.09±0.02b	0.0±0.0	0.0±0.0	92.00±2.65a	0.71±0.02a	3.67±1.45bc	0.09±0.02c
TUTGmGH3	21.00±1.53a	0.23±0.03a	5.00±1.00bc	0.13±0.02b	0.0±0.0	0.0±0.0	79.33±2.96b	0.93±0.01b	19.00±1.15a	0.80±0.03a
COMMERCIAL INOC	0.0±0.0	0.0±0.0	0.0±0.0	0.0±0.0	0.0±0.0	0.0±0.0	0.0±0.0	0.0±0.0	0.0±0.0	0.0±0.0
F-statistics	15.32***	1.64ns	61.82***	39.20***	3.66*	4.27*	333.62***	331.13***	41.04***	419.74***

Table S4: Nodule number and nodule dry matter of legume species used in cross-infectivity test in the glasshouse in 2022.

Treatments	Cowpea cv. IT10K-866-1		Bambara groundnut LR. SSD8		Kersting's groundnut LR. Dowie		Common bean cv. NUA 721		Soybean cv. TGX1937-1F	
	Nodule no. per plant	Nodule DM mg. plant -1	Nodule no. per plant	Nodule DM mg. plant -1	Nodule no. per plant	Nodule DM mg. plant -1	Nodule no. per plant	Nodule DM mg. plant -1	Nodule no. per plant	Nodule DM mg. plant -1
TUTVuSA1	7.33±0.88e	0.23±0.02e	0.0±0.0	0.0±0.0	0.0±0.0	0.0±0.0	0.0±0.0	0.0±0.0	25.67±1.20b	0.17±0.01b
TUTVuSA2	46.33±2.03b	1.28±0.02a	0.0±0.0	0.0±0.0	0.0±0.0	0.0±0.0	0.0±0.0	0.0±0.0	0.0±0.0	0.0±0.0
TUTVuSA3	23.67±1.33d	0.68±0.04d	0.0±0.0	0.0±0.0	14.33±1.20g	0.10±0.01b	0.0±0.0	0.0±0.0	0.0±0.0	0.0±0.0
TUTMgSA1	0.0±0.0	0.0±0.0	0.0±0.0	0.0±0.0	73.00±2.52c	0.95±0.36a	0.0±0.0	0.0±0.0	0.0±0.0	0.0±0.0
TUTMgSA2	0.0±0.0	0.0±0.0	0.0±0.0	0.0±0.0	35.67±1.76e	0.22±0.01b	0.0±0.0	0.0±0.0	0.0±0.0	0.0±0.0
TUTMgSA3	0.0±0.0	0.0±0.0	6.33±1.33d	0.07±0.01cd	22.33±2.19f	0.19±0.01b	0.0±0.0	0.0±0.0	8.33±0.33ef	0.15±0.00bc
TUTVsES1	9.33±1.45e	0.07±0.02f	26.00±2.08c	0.32±0.01cd	0.0±0.0	0.0±0.0	0.0±0.0	0.0±0.0	0.0±0.0	0.0±0.0
TUTVsES2	38.67±3.48c	0.80±0.01c	95.33±3.84b	0.71±0.22b	0.0±0.0	0.0±0.0	0.0±0.0	0.0±0.0	2.33±0.33g	0.03±0.01e
TUTVsES3	0.0±0.0	0.0±0.0	27.33±1.45c	0.32±0.01c	0.0±0.0	0.0±0.0	0.0±0.0	0.0±0.0	0.0±0.0	0.0±0.0
TUTPvES1	0.0±0.0	0.0±0.0	0.0±0.0	0.0±0.0	11.00±1.15g	0.10±0.00b	12.61±0.05c	0.0±0.0	0.0±0.0	0.0±0.0
TUTPvES2	0.0±0.0	0.0±0.0	10.33±0.67d	0.08±0.01cd	0.0±0.0	0.0±0.0	14.67±0.88b	0.12±0.01a	7.33±0.33f	0.09±0.01d
TUTPvES3	6.11±0.09e	0.0±0.0	0.0±0.0	0.0±0.0	28.33±1.20f	0.21±0.01b	18.00±2.08a	0.14±0.01a	11.33±0.88de	0.10±0.01d
TUTGmGH1	0.0±0.0	0.0±0.0	13.33±1.45d	0.13±0.01cd	0.0±0.0	0.0±0.0	6.33±0.88d	0.07±0.01b	13.67±1.45d	0.11±0.01d
TUTGmGH2	6.67±0.88e	0.08±0.01f	16.67±2.33cd	0.11±0.01cd	7.23±0.88H	02.0±0.03c	0.0±0.0	0.0±0.0	39.33±1.45a	0.27±0.01a
TUTGmGH3	20.33±1.45d	0.26±0.01e	5.33±0.67d	0.05±0.01d	7.23±0.88h	0.02±0.3c	3.33±0.33e	0.02±0.00c	13.67±0.88d	0.12±0.02cd
COMMERCIAL INOC	72.33±2.40a	1.03±0.01b	134.33±9.70a	1.35±0.09a	138.00±2.65a	1.25±0.02a	15.33±2.73b	0.12±0.02a	18.67±1.76c	0.17±0.01b
F-statistics	143.98***	508.00**	149.80***	28.45***	288.43**	13.74***	13.61***	12.69***	105.14***	33.89***

Table S4: Continue

Treatments	Velvet bean cv. IIHR PS 2		Jack bean cv. Accession 498		Pigeonpea cv. ICEAP00850		Mungbean cv. VC6 153 (B-20P)	
	Nodule no. per plant	Nodule DM mg. plant $^{-1}$	Nodule no. per plant	Nodule DM mg. plant $^{-1}$	Nodule no. per plant	Nodule DM mg. plant $^{-1}$	Nodule no. per plant	Nodule DM mg. plant $^{-1}$
TUTVuSA1	6.33±0.33fg	0.09±0.01c-e	0.0±0.0	0.0±0.0	10.00±1.15e	0.10±0.02e	0.0±0.0	0.0±0.0
TUTVuSA2	11.33±0.67de	0.10±0.01cd	6.33±0.88b	0.07±0.01b	4.33±0.33e	0.04±0.01e	0.0±0.0	0.0±0.0
TUTVuSA3	13.33±1.45cd	0.10±0.01c-e	0.0±0.0	0.0±0.0	0.0±0.0	0.0±0.0	0.0±0.0	0.0±0.0
TUTMgSA1	3.00±0.00g	0.03±0.00e	0.0±0.0	0.0±0.0	27.67±2.03d	0.23±0.02d	4.67±1.20d	0.03±0.01c
TUTMgSA2	4.33±2.03g	0.14±0.07bc	0.0±0.0	0.0±0.0	0.0±0.0	0.0±0.0	0.0±0.0	0.0±0.0
TUTMgSA3	84.33±2.73a	0.89±0.02a	2.33±0.67c	0.01±0.00c	0.0±0.0	0.0±0.0	5.00±1.00c	0.07±0.01b
TUTVsES1	0.0±0.0	0.0±0.0	2.33±0.88c	0.01±0.01c	0.0±0.0	0.0±0.0	0.0±0.0	0.0±0.0
TUTVsES2	0.0±0.0	0.0±0.0	6.67±0.88b	0.06±0.01b	0.0±0.0	0.0±0.0	0.0±0.0	0.0±0.0
TUTVsES3	0.0±0.0	0.0±0.0	2.33±0.33c	0.01±0.00c	0.0±0.0	0.0±0.0	0.0±0.0	0.0±0.0
TUTPvES1	0.0±0.0	0.0±0.0	0.0±0.0	0.0±0.0	0.0±0.0	0.0±0.0	7.67±0.88b	0.07±0.01b
TUTPvES2	16.67±0.67bc	0.14±0.00bc	10.33±0.88a	0.11±0.00a	7.33±0.88e	0.05±0.01e	0.0±0.0	0.0±0.0
TUTPvES3	5.33±0.88fg	0.07±0.33c-e	0.0±0.0	0.0±0.0	0.0±0.0	0.0±0.0	0.0±0.0	0.0±0.0
TUTGmGH1	5.67±0.67fg	0.03±0.01de	0.0±0.0	0.0±0.0	90.33±1.86a	0.68±0.02b	8.33±1.45b	0.06±0.01bc
TUTGmGH2	12.00±1.15de	0.14±0.01bc	0.0±0.0	0.0±0.0	39.33±2.60c	0.34±0.02c	0.0±0.0	0.0±0.0
TUTGmGH3	5.33±0.67fg	0.04±0.01de	0.0±0.0	0.0±0.0	74.00±3.21b	0.85±0.04a	22.67±1.20a	0.34±0.01a
F-statistics	326.23**	100.88**	17.78***	55.63***	302.73***	248.04***	40.86***	167.64***
